# *Poecilobdella manillensis* Bioactive Peptides Reduce Oxidative Stress and Regulate Metabolic Reprogramming via the IIS/FOXO Pathway to Improve Hypoxic Injury

**DOI:** 10.3390/antiox15080936

**Published:** 2026-07-28

**Authors:** Jiahui Wang, Jieshu Li, Shuqi Li, Jinze Li, Zichen Lei, Jinchai Qi, Gengyang Liu, Zekun Yu, Yueying Yuan, Jing Han, Tao Ma, Yonggang Liu

**Affiliations:** 1 School of Chinese Materia Medica, Beijing University of Chinese Medicine, Beijing 100029, China; 20250941567@bucm.edu.cn (J.W.); 20240935080@bucm.edu.cn (J.L.); 20230935068@bucm.edu.cn (S.L.); 20250935106@bucm.edu.cn (J.L.); 20250935290@bucm.edu.cn (Z.L.); 20230941471@bucm.edu.cn (J.Q.); 20240935148@bucm.edu.cn (G.L.); 20240935147@bucm.edu.cn (Z.Y.); 2 Research Institute of Chinese Medicine, Beijing University of Chinese Medicine, Beijing 102488, Chinahanj@bucm.edu.cn (J.H.); 3 Linyi Key Laboratory of TCM Mechanism Analysis and Health Product Transformation, Linyi 276000, China

**Keywords:** IIS/FOXO pathway, oxidative stress, metabolic reprogramming, hypoxic injury, PMP

## Abstract

FOXO/DAF-16 is involved in stress resistance and metabolic regulation, but the molecular mechanisms of its interaction with hypoxia remain unclear. This study aimed to evaluate the anti-hypoxic effects of *Poecilobdella manillensis* bioactive peptide (PMP) and to investigate whether IIS/FOXO acts as a key node in mediating the regulation of oxidative stress and metabolic reprogramming. In the chemical hypoxia model of *Caenorhabditis elegans* (*C. elegans*) induced by sodium sulfite, PMP treatment improved the survival status and movement, feeding, and reproductive ability of hypoxic *C. elegans*, and significantly increased their survival rate. It also reduced reactive oxygen species (ROS) and lipofuscin levels in *C. elegans*, enhancing their tolerance to oxidative and heat stress. In the terminal normobaric hypoxia mice model, PMP intervention prolonged the survival time of hypoxic mice, alleviated the damage of heart, lung, and brain tissues, and increased superoxide dismutase (SOD) activity and glutathione (GSH) levels, and decreased malondialdehyde (MDA) concentrations and lactate dehydrogenase (LDH) activity in serum and tissues of mice. ^1^H-NMR metabolomics analysis showed that PMP treatment reversed hypoxia-induced abnormalities in key metabolites such as glucose, lactic acid, glutamic acid, and taurine. Next, we utilized *C. elegans* mutants deficient in *daf-2*, *age-1*, *akt-1*, *daf-16*, and *hsp-16.2,* and further observed the nuclear translocation of DAF-16 in *DAF-16::GFP C. elegans*. The results showed PMP induced DAF-16 nuclear translocation and upregulated the expression of downstream SOD-3. Key metabolites representing antioxidant and energy metabolism were measured in the *daf-16* mutant *C. elegans*. The results showed that PMP intervention failed to restore the levels of glucose, lactic acid, glutamic acid, and taurine in the mutant. Finally, 12 peptides containing antioxidant-related bioactive amino acid residues in PMP were screened by UPLC-Q-Exactive-MS and peptide biological activity prediction. Among them, molecular docking showed that KPPGP had a good binding with FOXO1. In conclusion, in *C. elegans*, PMP activated DAF-16/FOXO by inhibiting the Insulin/insulin-like growth factor-1 signaling (IIS) pathway and regulated redox homeostasis and metabolic reprogramming to resist hypoxia injury, and this protective effect was also observed in mouse models. IIS/FOXO can be used as a key node to regulate oxidative stress and energy metabolism under hypoxic conditions, and the identification of KPPGP provides insights into the screening and study of bioactive peptides in natural products.

## 1. Introduction

Oxygen is vital to all organisms, playing a role in the survival and function of cells [1]. Hypoxia is defined as a lack of oxygen in the body or specific tissues, and is a hallmark of many diseases, such as ischemic stroke, hypertension, and chronic obstructive pulmonary disease. Under hypoxic conditions, energy metabolism in tissue cells is impaired, redox homeostasis is imbalanced, ROS accumulate excessively, and lipid peroxidation occurs, ultimately leading to cellular damage, tissue necrosis, organ failure, and even death. Hypoxia is classified into acute and chronic intermittent hypoxia. Both of them increase the level of ROS in chemoreceptor cells, inhibit voltage-gated K^+^ channels, cause cell depolarization, Ca^2+^ influx, and release of excitatory neurotransmitters. In addition, chronic hypoxia contributes to the central nervous system inflammation and cardiopulmonary dysfunction [2], which interferes with critical cellular signaling and physiological processes.

Hypoxia affects the respiratory chain by Complex I and Complex III. Insufficient supply of the terminal electron acceptor in the electron transport chain leads to electron leakage at Complex I and Complex III, where O_2_ is reduced to O_2_^−^·, and ROS such as H_2_O_2_ and ·OH are generated in this process [3]. Excessive ROS production leads to oxidative stress and damages to lipids, proteins, and nucleic acids [4]. Complex III plays an irreplaceable role in hypoxia-induced ROS production. After inhibiting Rieske iron-sulfur protein expression of complex III by RNAi, hypoxia-induced HIF-1α and ROS are significantly reduced [5]. HIF-1α regulates mitochondrial metabolism and biogenesis to inhibit mitochondrial ROS production, and promotes glycolysis to maintain energy supply [6]. The energy substrate shortage causes an abnormal cell metabolism which is a significant factor in hypoxia injury. Hypoxia promotes glycolysis through HIF-1α-driven metabolic reprogramming, which reduces oxygen dependence of tumor cell and supports tumor cell growth and immune escape [7]. NAD metabolism coordinates the cellular hypoxia response by interactions with HIFs [8]. As a pivot of oxidative stress and energy metabolism disorders, NADH reduction stress significantly influences glucose, lipid, amino acid, and nucleotide metabolic pathways [9]. Hypoxia-induced NADH reduction stress increases oxidative damage and energy collapse by enhancing ROS production and reducing ATP. Growing evidence shows the bidirectional regulatory relationship between redox homeostasis and metabolic reprogramming. Both reduction and oxidation can induce metabolic reprogramming of vascular cells. Under hypoxia reduction stress, the accumulation of L-2-hydroxyglutaric acid in cells attenuates glycolysis and mitochondrial respiration to protect cells [10]. Intervention strategies targeting redox stress and metabolic reprogramming under hypoxic conditions are essential for understanding various hypoxia-related pathologies and diseases.

The interaction between HIF-1α and stress signaling pathways such as PI3K/Akt, MAPK, Nrf2, and NF-κB has enriched the understanding of hypoxic microenvironments [6]. However, hypoxia-induced cellular damage involves not only the activation of acute cascade stress signals and redox imbalance, but is also accompanied by a cellular metabolic crisis caused by disruption of energy substrate supply. This phenomenon suggests that the origin of signaling axis activation is not limited to the non-classical pathway induced by hypoxia, but may be more likely directly governed by the nutrition sensing and growth regulation networks. The IIS pathway is a highly conserved nutritional sensing and signal transduction pathway. It has been shown to alleviate oxidative stress by regulating downstream transcription factors FOXO/DAF-16 [11,12]. The KEGG pathway database clearly lists “oxidative stress resistance” as one of the core functions of FOXO/DAF-16 transcription factors [13]. It is worth emphasizing that the target genes activated by FOXO/DAF-16 are not involved in a single stress response or metabolic reaction, but rather constitute a synergistic regulatory network across multiple dimensions covering energy metabolism, glucose metabolism, lipid metabolism, etc. [12,14]. Although previous studies have confirmed that the IIS pathway changes under hypoxic conditions [15], the relationship with hypoxic injury remains unclear. Understanding the role of the IIS pathway in the regulatory network of hypoxic injury will help reveal how cells maintain homeostasis and survival under the dual pressures of oxidative stress and energy deprivation.

Chemical medicines such as nimodipine [16] and dexamethasone [17], as well as interventions like oxygen therapy, have a certain relieving effect on hypoxic symptoms. However, significant side effects and severe adverse reactions exist. Therefore, safer and more effective anti-hypoxic substances are of great scientific importance. Most bioactive peptides are composed of 2–20 amino acid residues and have independent biological functions (e.g., antioxidation) [18]. Due to their good biocompatibility [19], excellent resistance to gastrointestinal digestion at low concentrations, non-toxicity, and targeting effect, there is potential in the drug discovery of novel bioactive peptides [20,21]. The dried body of the medicinal leech *P. manillensis* has anticoagulation, antioxidation, and anti-inflammatory effects, and is widely used in the clinical treatment of stroke. According to previous research, leech extract significantly enhances tissue tolerance to hypoxia [22]. *P. manillensis* shows considerable potential to be further explored in resisting hypoxia injury.

Under hypoxic conditions, imbalances in redox homeostasis and energy metabolism disorders jointly drive tissue injury. The IIS pathway undergoes alterations in the hypoxic environment and may participate in the regulatory mechanism against hypoxia. FOXO/DAF-16 plays a key regulatory role in multiple physiological processes such as oxidative stress resistance, metabolism, and energy generation, and may serve as a core node for integrating redox and metabolic signals to coordinate oxidative stress and energy metabolism disorders under hypoxic conditions. This study evaluated the anti-hypoxia effect of PMP, elucidated whether it coordinated oxidative stress and metabolic disorders under hypoxia through IIS/FOXO, and preliminarily identified peptides in PMP that potentially interact with FOXO1. The study will provide new insights into the regulatory mechanisms of oxidative stress and energy metabolism under hypoxia, and provide a reference for the screening and study of bioactive peptides from natural origins.

## 2. Materials and Methods

### 2.1. Materials

*P. manillensis* freeze-dried powder (Yunnan Century Huabao Pharmaceutical Industry De-velopment Co., Ltd.) (Chuxiong, China); Anhydrous sodium sulfite (Tianjin Damao Chemical Partnership Enterprise) (Tianjin, China); Rhodiola Ginseng Capsules (RGC, Ingredients: Rhodiola extract and ginseng extract; Jiangxi Renhe Pharmacy Co., Ltd.) (Zhangshu, China); LDH Assay Kit, H2DCFDA ROS fluorescent probe (Nanjing Jiancheng Biological Engineering Institute) (Nanjing, China); Total SOD Activity Assay Kit, MDA Assay Kit, GSH and GSSG Assay Kit (Shanghai Beyotime Biotechnology Co., Ltd.) (Shanghai, China).

### 2.2. Animals

Wild-type N2, DR26 *daf-16* (*m26*), CF1553 (*muIs84* [(*pAD76*) *sod-3::GFP*)]), TJ356 (*zIs356* [*daf-16p::daf-16a/b::GFP+rol-6*]), VC475 [ *hsp-16.2* (*gk249*)], CB1370 *daf-2* (*e1370*) III, TJ1052 *age-1* (*hx546*), RB759 *akt-1* (*ok525*) V, *E. coli* OP50 were purchased from the Caenorhabditis Genetics Center (Minneapolis, MN, USA); female Kunming mice (8 weeks old, body weight 18~22 g) were purchased from SPF (Beijing) Biotechnology Co., Ltd. (Beijing, China) (approval No. SCXK (Beijing) 2019-0010), and housed in the SPF-grade animal facility of the Beijing University of Chinese Medicine Experimental Animal Center under environmental conditions of a 12 h dark cycle, constant temperature of 22 ± 2 °C, and constant humidity of 50 ± 5%, with free access to food and water. All animal experiments strictly adhered to the National Research Council’s Guidelines for the Care and Use of Laboratory Animals and were approved by the Animal Ethics Committee of Beijing University of Chinese Medicine (ethics number: BUCM-1-2024060501-1036, approval date: 5 June 2024).

### 2.3. Extraction and Purification of PMP

*P. manillensis* freeze-dried powder was degreased with 4 times *v*/*w* petroleum ether using ultrasound for 1 h. After discarding the petroleum ether and evaporating the residual solvent, the residue was extracted twice with 15 times *v*/*w* of 4.3% NaCl solution (each extraction: 2 h ultrasonication, total 4 h). The combined extracts were diluted with pre-cooled anhydrous ethanol to obtain 60% (*v*/*v*) ethanol solution. Ethanol solution was kept at 4 °C for 6 h, and centrifuged at 8000 r·min^−1^ for 20 min at 4 °C. The supernatant was collected, and the precipitate was dissolved in an equal volume of water to be centrifuged again, and this dissolution–centrifugation cycle was repeated three times. All supernatants were freeze-dried. The extraction yield of PMP was approximately 4%.

### 2.4. Study on C. elegans

#### 2.4.1. Construction of Chemical Hypoxia Model of *C. elegans* with Sodium Sulfite

L4-stage *C. elegans* were transferred to 10 mL glass test tubes. For the model group and treatment group, the final volume of each tube was adjusted to 1 mL using a 2.0 g∙L^−1^ sodium sulfite solution; the control group used an equal volume of sterile deionized water. Sealed glass test tubes were placed in a 22 °C or 16 °C biochemical incubator. Following hypoxia exposure, *C. elegans* were transferred to fresh FuDR-containing medium inoculated with *E. coli* OP50 and allowed to recover for 24 h at the corresponding temperature.

#### 2.4.2. Determination of *C. elegans* Survival Rate

After hypoxia-reoxygenation treatment of N2 *C. elegans* (>200), a platinum wire probe was used to lightly touch the *C. elegans*, and no autonomic movement response within 10 s was recorded as death. Survival rates were calculated for each group, and the experiment was repeated three times independently.

#### 2.4.3. Determination of the *C. elegans* Head Swing Frequency

After exposure to hypoxia and reoxygenation, 20 *C. elegans* from each group were randomly selected and transferred onto standard NGM culture medium. M9 buffer was then added, followed by a 1 min equilibration period. The number of head–tail swings of *C. elegans* in each group observed under the microscope within 20 s was recorded, and the experiment was repeated three times independently.

#### 2.4.4. Determination of *C. elegans* Swallow Frequency

After exposure to hypoxia and reoxygenation, 20 *C. elegans* from each group were randomly selected and placed under a microscope. The pharyngeal pump frequency of *C. elegans* in each group within 20 s was recorded, and the experiment was repeated three times independently.

#### 2.4.5. Determination of *C. elegans* Egg Laying

After exposure to hypoxia and reoxygenation, 10 *C. elegans* were randomly selected from each group and placed individually on standard NGM medium coated with *E. coli* OP50. Cultures were maintained at 22 °C in a biochemical incubator. *C. elegans* were transferred daily to fresh standard NGM medium until egg-laying activity ceased. Total eggs laid per worm were counted for each group. The experiment was repeated three times independently.

#### 2.4.6. Determination of *C. elegans* Locomotor Ability

After exposure to hypoxia and reoxygenation, 30 *C. elegans* were randomly selected from each group and observed under a microscope. Each *C. elegans* was lightly touched with a platinum wire probe, and its locomotion was classified as follows: Type A, individuals exhibiting at least one complete sinusoidal movement cycle without external stimulation; Type B, those initiating sinusoidal movement only after mild tail stimulation with the probe; Type C, those failing to produce effective sinusoidal waves even after repeated stimulation (3 times within 10 s), displaying only uncoordinated movements such as head swaying or tail tremors. The motility distribution was recorded for each group, and the experiment was independently repeated three times.

#### 2.4.7. Measurement of *C. elegans* Body Length

L4-stage *C. elegans* were randomly divided into control and experimental groups and transferred to NGM plates seeded with *E. coli* OP50 or NGM plates supplemented with different concentrations of PMP. After 24 h, these *C. elegans* were washed three times with M9 buffer, centrifuged, and the supernatant was discarded. The worm pellet was resuspended in 20 μL of anesthetic solution, placed on a clean glass slide, and imaged under bright-field illumination on an inverted fluorescence microscope. Body length was measured using NIS-Elements (AR 6.20.00) image analysis software. A total of 60 *C. elegans* were examined per group in each independent experiment, and the experiment was repeated three times independently.

#### 2.4.8. Measurement of ROS Levels in *C. elegans*

After hypoxia-reoxygenation, N2 *C. elegans* in each group were incubated with 100 μL of 10 μM H_2_DCFDA at 22 °C for 2 h. These *C. elegans* were then rinsed, pelleted by centrifugation, and the supernatant was discarded. The pellet was resuspended in 20 μL of anesthetic solution, transferred to a glass slide, and imaged under an inverted fluorescence microscope (excitation 480 nm, emission 530 nm). Fluorescence intensity was quantified using ImageJ 1.54p. A total of 60 *C. elegans* per group were analyzed in each independent experiment, and the experiment was repeated three times independently.

#### 2.4.9. Determination of Lipofuscin Levels in *C. elegans*

After hypoxia-reoxygenation, N2 *C. elegans* in each group were resuspended in 20 μL of anesthetic solution, transferred to a glass slide, and imaged under an inverted fluorescence microscope (excitation 480 nm, emission 530 nm). Fluorescence intensity was quantified using ImageJ. A total of 60 *C. elegans* per group were analyzed in each independent experiment, and the experiment was repeated three times independently.

#### 2.4.10. Determination of Survival Rate of *C. elegans* Under Oxidative Stress

Following hypoxic-reoxygenation treatment, 120 *C. elegans* were randomly selected from each group and transferred to H_2_O_2_-containing medium. Survival status was recorded hourly until all *C. elegans* died. The experiment was repeated three times independently.

#### 2.4.11. Determination of Survival Rate of *C. elegans* Under Heat Stress

Following N2 *C. elegans* hypoxia-reoxygenation treatment, 120 *C. elegans* were randomly selected from each group and transferred to standard NGM medium. Cultured at 37 °C, survival status was recorded hourly until all *C. elegans* died. The experiment was repeated three times independently.

#### 2.4.12. Measurement of *C. elegans* Lifespan

Following hypoxic-reoxygenation treatment of N2 *C. elegans*, the experimental day was designated as Day 0. Thereafter, *C. elegans* were transferred to fresh plates with identical formulations every two days to maintain culture conditions. Daily survival assessments were conducted by gently touching the head and body of each *C. elegans* with a platinum wire at fixed intervals. The number of surviving and deceased individuals in each group was recorded simultaneously until all *C. elegans* died. In each independent experiment, 120 *C. elegans* were detected in each group of samples. The experiment was repeated three times independently.

#### 2.4.13. NMR Metabolomics Analysis

##### NMR Sample Preparation

Following hypoxia-reoxygenation treatment of N2 *C. elegans*, *C. elegans* were washed with M9 buffer three times, then washed with deionized water three times. A 600 μL volume of pre-chilled methanol solution (methanol:water = 2:1, *v*/*v*) was added to the *C. elegans* sample and homogenized for 4 min. After homogenization, the sample was stored at −20 °C for 60 min. The sample was centrifuged at 12,000 rpm at 4 °C for 20 min, and the supernatant was collected. Another 600 μL of pre-chilled methanol solution was added, and the process was repeated again. The extract was extracted three times, then the supernatant was combined. Methanol in the supernatant was removed by the nitrogen blowdown apparatus, then the residue was freeze-dried. Afterward, 400 μL of deuterated water and 40 μL of phosphate buffer were added to the dried powder, and the sample was sonicated for 5 min. After centrifuging at 12,000 rpm at 4 °C for 20 min, the supernatant was collected, transferred to an NMR tube, and analyzed. Six independent samples were analyzed per group.

##### NMR Data Acquisition

NMR data for *C. elegans* were acquired using a Bruker Avance III 600 MHz NMR spectrometer (Bruker Corporation, Billerica, MA, USA) equipped with an ultra-low temperature probe. The sample test sequence was completely randomized to eliminate systematic operational deviations. The NOESY PR1D pulse sequence was employed under the following conditions: temperature 298 K, relaxation delay time 2 s, spectral width 12,500 Hz, and number of scans 512.

##### Data Processing and Statistical Analysis

^1^H-NMR spectra were processed using Mest ReNova 14.0 software, with TSP serving as the internal standard for chemical shift reference. The integration interval was set from δ 0.60~10.0, excluding the water peak at δ 4.50~5.50, with δ 0.01 as the integral area. The integrated spectral data underwent area normalization. Data processed by Mest ReNova 14.0 were imported into SIMCA 14.0 software for multivariate statistical analysis following adaptive transformation. PCA was employed to detect separation trends and outliers between experimental groups; OPLS-DA was used to assess model validity and identify differential metabolites. Differential metabolites were screened based on VIP > 1 and *p* < 0.05. The Benjamini–Hochberg method was used for FDR correction. Relative peak area integration was performed for differential metabolites, excluding overlapping peaks from statistical analysis. Relative peak area data for differential metabolites underwent statistical processing. Metabolite identification was conducted via a literature review of databases such as HMDB, http://www.hmdb.ca/ (accessed on 27 July 2025), BMRB, https://bmrb.io/ (accessed on 27 July 2025) and so on. Metabolic pathway analysis was performed using https://www.metaboanalyst.ca/home.xhtml (accessed on 10 August 2025). Both the influence factor threshold and *p*-value threshold were set to 0.1. Pathways with an influence factor > 0.1 and *p*-value < 0.1 were identified as pathways associated with differential metabolites.

#### 2.4.14. Measurement of DAF-16 and SOD-3 Protein Expression in Hypoxic *C. elegans*

TJ356 (*zIs356* [*daf-16p:: daf-16a/b::GFP+rol-6*]) and CF1553 (*muIs84* [(*pAD76*) *sod-3:: GFP*)]) *C. elegans* were subjected to hypoxia-reoxygenation treatment, followed by addition of 20 μL anesthetic solution. Anesthetized *C. elegans* were transferred to clean microscope slides. Fluorescence images were acquired using an inverted fluorescence microscope at an excitation wavelength of 480 nm and an emission wavelength of 530 nm. Quantitative analysis of fluorescence images was performed using ImageJ software. Each independent experiment analyzed 100 *C. elegans* per group, with the experiment repeated three times independently.

### 2.5. Study on Mice

#### 2.5.1. Determination of Survival Time of Mice Exposed to Terminal Normobaric Hypoxia

Mice were adaptively fed for 7 days and randomly divided into the control, model, RGC, and PMP group by the random number table method, and there were 10 mice in every group. RGC was used as the positive control. The dosage of RGC is 266.6 mg·kg^−1^, based on the human daily recommended dosage converted to mouse body weight, and that of PMP is 400.0 mg·kg^−1^. Mice in each group received daily oral administration of equal-volume physiological saline or corresponding drugs (10 mL∙kg^−1^) for 7 consecutive days. Behavioral status and body weight changes in mice were observed daily. One hour after the final administration, the control group was not exposed to a hypoxic environment to establish the normal baseline of each index, and other groups were exposed to a normobaric hypoxia environment: each mouse was individually placed in a 250 mL ground-mouth jar containing 5 g sodium lime (CO_2_ absorption with lime to avoid superposition of systemic acidosis), with the jar mouth sealed using petroleum jelly. The experimental procedures strictly adhered to animal ethics guidelines. Starting from the sealing of the tank mouth, the state changes in the mice were closely observed. The survival time was defined as the time from the beginning of sealing until sign of terminal distress (convulsion and coma) in mice. Upon reaching this endpoint, the mice were immediately anesthetized with inhaled isoflurane. Mice with accidental death or abnormal status during the experiment were excluded from the final statistical analysis. All animals in this experiment completed the study as planned, with no exclusions. In the hypoxia survival time determination assay, the personnel recording survival times were unaware of the grouping.

#### 2.5.2. Detection of Organ Index, Histopathology and Oxidative Stress in Mice Exposed to Terminal Normobaric Hypoxia

After the end of terminal normobaric hypoxia exposure, the mice were anesthetized with inhaled isoflurane, blood was collected from the femoral vein, and heart, lung, and brain tissues were collected. Blood samples were centrifuged at 3000 r·min^−1^ at 4 °C for 10 min to extract serum. The organs were washed, dried, and weighed with pre-cooled normal saline, and the organ index of heart, lung, and brain (organ weight/body weight, mg/g) was calculated. Half of the tissues were fixed with 4% paraformaldehyde solution, followed by routine paraffin embedding, sectioning, hematoxylin-eosin staining, microscopic examination, image acquisition, and analysis. The other half was frozen in liquid nitrogen and stored at −80 °C, and used together with the serum for the determination of SOD, MDA, LDH, and GSH.

### 2.6. Identification of PMP Using UPLC-Q-Exactive-MS

#### 2.6.1. Sample Preparation

A total of 10 mg of PMP was dissolved in 2.5 mL of ultrapure water and an equal volume of 0.2% TFA solution. The sample was loaded onto an SPE column pretreated with 5 mL acetonitrile and 5 mL 0.1% TFA solution, and eluted with 6 mL of 0.1% TFA solution to remove salts and polar interferences. Then the sample was eluted with 6 mL of 40% acetonitrile in 0.1% TFA solution to release peptides. The nitrogen blowdown apparatus was used to remove the acetonitrile in the eluate, and a freeze dryer was used to dewater the sample. Finally, the powder was resuspended for LC-MS/MS analysis.

#### 2.6.2. Chromatographic and Mass Spectrometry Conditions

The UPLC separation was performed on an ACQUITY BEH C18 chromatography column (100 mm × 2.1 mm, i.d., 1.7 µm, Waters, Milford, MA, USA). The mobile phase consisted of two solvents: (A) 0.1% formic acid aqueous solution and (B) 98% acetonitrile (1% formic acid). The metabolites were separated using the following gradient elution profile: 0~3 min; 97% A; 3~83 min; 97% A~80% A; 83~108 min; 80% A~60% A; 108~118 min; 60% A~5% A; 118~123 min; 5% A. The flow rate was 1.00 mL/min, with an injection volume of 10 µL, and the column temperature was maintained at 40 °C. After chromatographic separation, the mobile phase was introduced directly into the Q Exactive Plus combined quadrupole Orbitrap mass spectrometer coupled to the UPLC system (Thermo Fisher Scientific, Waltham, MA, USA). The MS system was operated in positive ion mode, with the mass range set at *m*/*z* 150–2000 Da at full scan resolution. The operating MS conditions were as follows: HESI; aux gas heater temp, 400 °C; spray voltage, +3.5 kV; capillary temp, 320 °C; aux gas heater temp, 400 °C; scanning mode, Full MS (Resolution 70,000)/dd-MS^2^ (Resolution 17,500); normalized collision energy, 20, 40, 60 eV.

#### 2.6.3. Data Analysis

Data spectrum analysis was performed using Qual Browser of Xcalibur software 4.1 (Thermo Fisher Scientific, USA). Peaks Studio software 13.1 was employed to analyze MS/MS data from raw spectrum files. For de novo sequencing, the parent ion mass error tolerance was set to 10.0 ppm, and the fragment mass error tolerance to 0.02 Da. The digestion method was selected as “no enzyme”. Neither fixed nor variable modifications were selected. The confidence threshold of de novo model is ≥70.0%, with all other parameters set to default values.

#### 2.6.4. Prediction of Possible Biological Activity of Peptides 

To evaluate the possible biological activities of the identified peptides, we first predicted their bioactivity using Peptide Ranker, with a screening threshold of ≥0.8. The BIOPEP-UWM database (https://www.uwm.edu.pl/biochemia/index.php/pl/biopep) (accessed on 9 September 2025) was used to identify peptides containing biologically active amino acid residues related to antioxidants. For peptides containing biologically active amino acid residues related to antioxidants, Innovagen online tools (https://pepcalc.com/) (accessed on 10 September 2025) were used to assess water solubility. Expasy tools (https://web.expasy.org/protparam/) (accessed on 10 September 2025) predicted stability. Peptides with an instability index <40 were considered stable, while those >40 were deemed unstable. The ToxinPred platform (https://webs.iiitd.edu.in/raghava/toxinpred/) (accessed on 10 September 2025) was employed to predict potential toxicity. The AdmetSAR system (http://lmmd.ecust.edu.cn/admetsar2) (accessed on 11 September 2025) assessed human intestinal absorption (HIA) properties.

#### 2.6.5. Molecular Docking

The 3D crystal structure of the FOXO1 protein (PDB ID: 6QZR) was downloaded from the PDB database, and the receptor protein was pretreated with Pymol software 3.1.6.1 to remove water molecules, small-molecule ligands, and heteroatoms. The 3D structure of KPPGP was constructed by the RPBS online server. AutoDockTools 1.5.6 was used to hydrogenate the receptor protein and peptide ligand, distribute the charge, and perform molecular docking and calculate the binding energy.

### 2.7. Statistical Analysis

Statistical analysis was performed using IBM SPSS Statistics 25 software. For comparisons between two groups, the independent-sample *t*-test was used if the data were normally distributed with homogeneous variances. Otherwise, the Wilcoxon rank-sum test was used. For comparisons involving multiple groups, the normality test was first conducted. If the conditions were met, ANOVA was used (homogeneity of variance, using the LSD post hoc test; if the variance is not uniform, Tamhane’s T2 test is used). Otherwise, the Wilcoxon rank-sum test was utilized (Kruskal–Wallis test). Survival and stress tolerance curves were plotted using Kaplan–Meier, and the log-rank test was used to compare the differences between groups. *p* < 0.05 was considered the threshold of significant difference. Graphics were drawn using GraphPad Prism 9.0 and Origin 2022 software.

## 3. Result

### 3.1. PMP Treatment Improves the Survival and Functional Status of Hypoxic C. elegans

**Figure 1:**
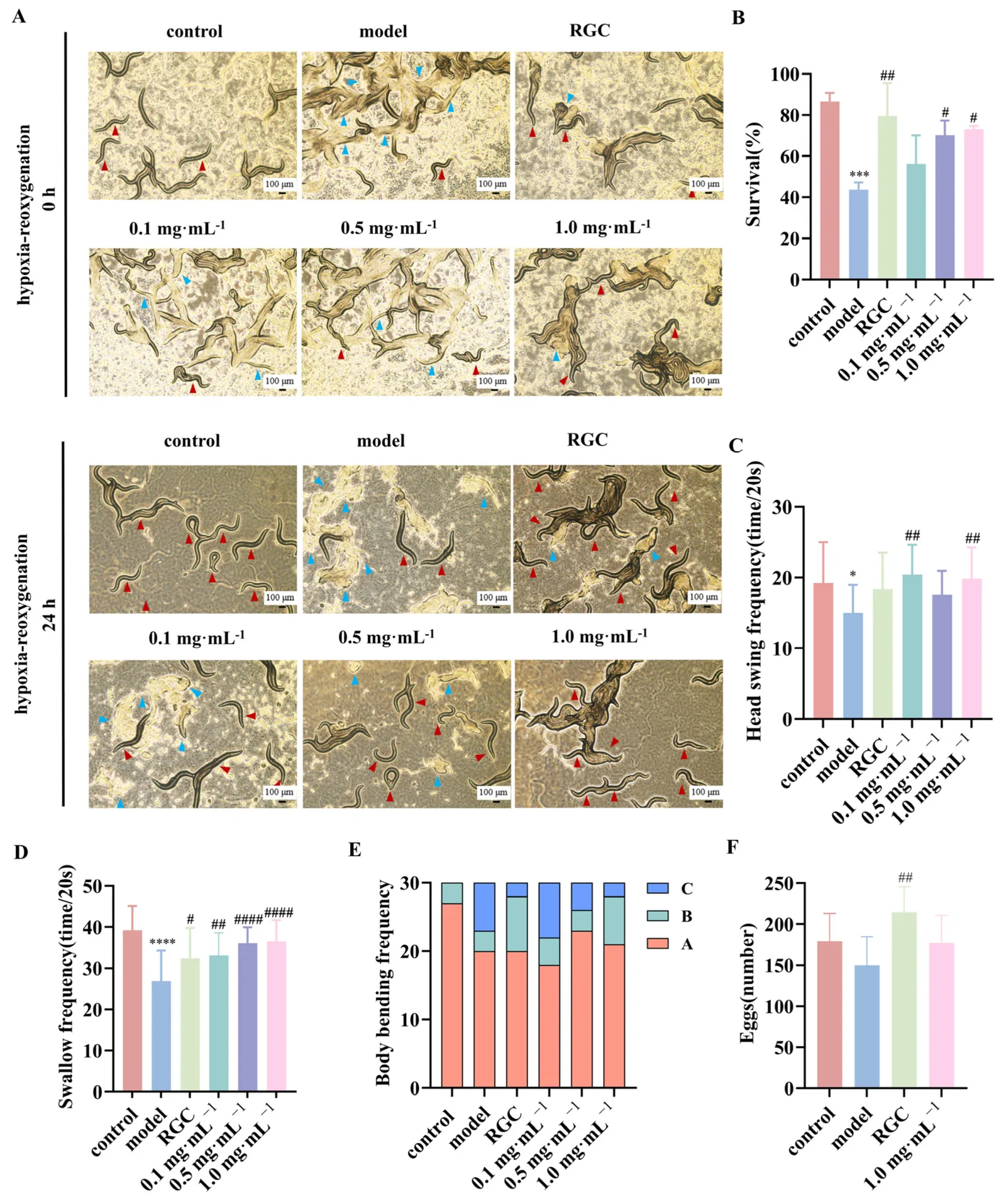
PMP treatment improves the survival and functional status of hypoxic *C. elegans*. (**A**) The survival diagram of *C. elegans* 0 h and 24 h after hypoxia-reoxygenation (survival: red triangle; death: blue triangle). Effect of PMP treatment on the survival rate (>200) (**B**), head swing frequency (*n* = 20) (**C**), swallow frequency (*n* = 20) (**D**), body bending frequency (*n* = 30) (**E**), and egg production (*n* = 10) (**F**) of hypoxic *C. elegans*. (Note: Compared with the control group, * *p* < 0.05, *** *p* < 0.001, **** *p* < 0.0001; compared with the model group, ^#^ *p* < 0.05, ^##^ *p* < 0.01, ^####^ *p* < 0.0001.)

The anti-hypoxia activity of PMP was evaluated preliminarily using the chemical hypoxia model of *C. elegans* induced by sodium sulfite (Figure S1). The protective effects of PMP treatment on hypoxic *C. elegans* were examined using concentrations of 0.1, 0.5, and 1.0 mg·mL^−1^ (Figure S2). The results showed that 0.5 and 1.0 mg·mL^−1^ PMP treatment significantly improved the survival status of hypoxic *C. elegans* and increased their survival rate (*p* = 0.005, 0.002) (Figure 1A,B). Meanwhile, 1.0 mg·mL^−1^ PMP intervention significantly increased the head–tail swing and pharyngeal pump frequency of hypoxic *C. elegans* (*p* = 0.0048, <0.0001), thereby improving their movement and feeding ability (Figure 1C,D). The results of exercise behavior analysis showed that after PMP intervention, the number of type A and type B nematodes increased, and the number of type C nematodes decreased (Figure 1E). In addition, the reproductive capacity of hypoxic *C. elegans* was also improved by PMP intervention (Figure 1F). PMP intervention reduced the damage of hypoxia to *C. elegans* and maintained their movement, feeding, and reproductive functions.

### 3.2. Alleviating Oxidative Stress Injury of Hypoxic C. elegans Improves the Body’s Stress Resistance

**Figure 2:**
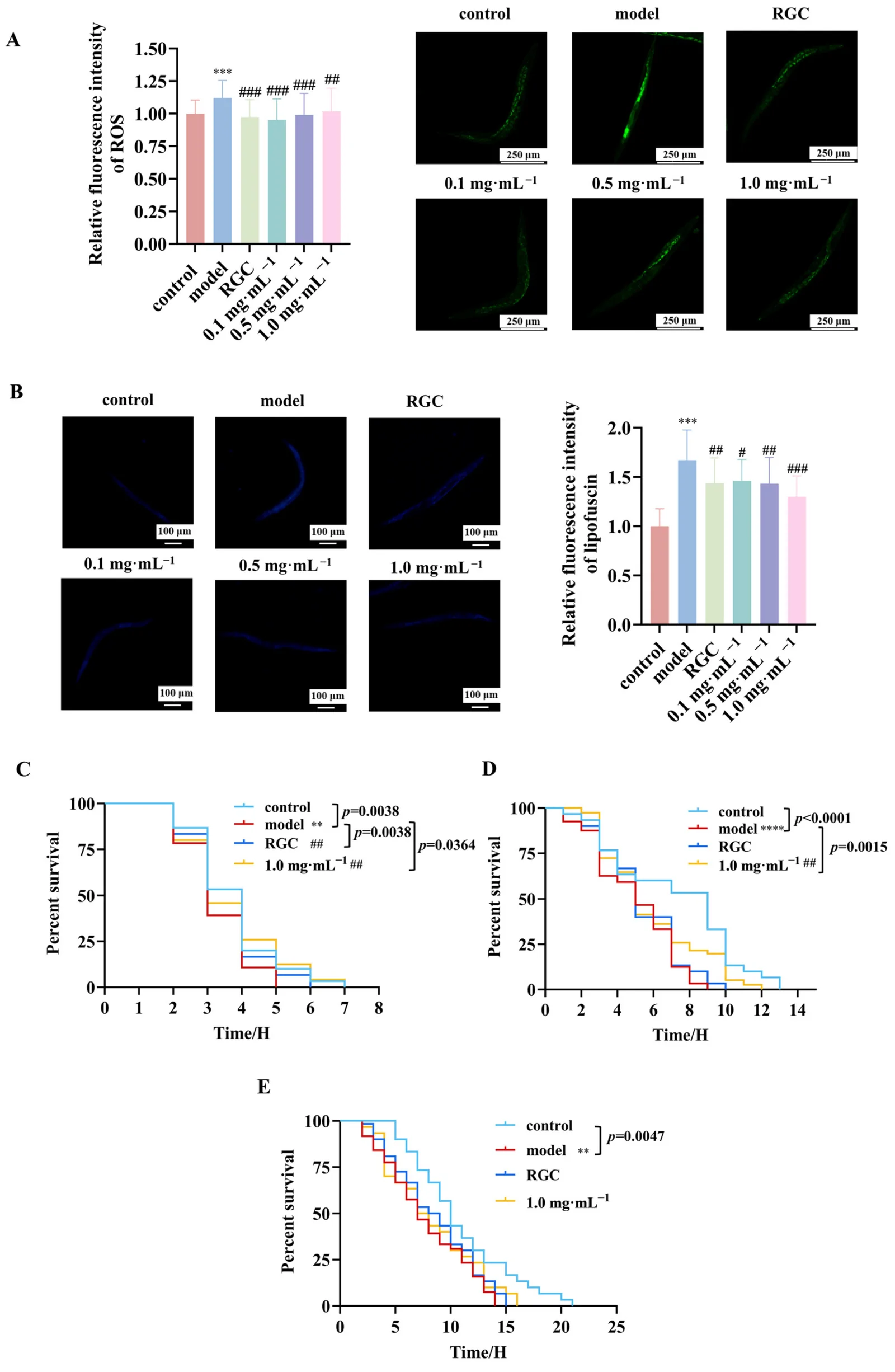
Alleviating oxidative stress injury of hypoxic *C. elegans* improve the body’s stress resistance. Effect of PMP treatment on the ROS level (*n* = 60) (**A**), lipofuscin level (*n* = 60) (**B**), and lifespan (*n* = 120) (**E**) in hypoxic *C. elegans*. The effect of PMP treatment on the survival rate of hypoxic *C. elegans* under oxidative stress (*n* = 120) (**C**) and heat stress (*n* = 120) (**D**). (Note: Compared with the control group, ** *p* < 0.01, *** *p* < 0.001, **** *p* < 0.0001; compared with the model group, ^#^ *p* < 0.05, ^##^ *p* < 0.01, ^###^ *p* < 0.001.)

Excessive activation of oxidative stress is a driving force of hypoxia-induced organismal damage [23]. Based on the aforementioned overall anti-hypoxic injury effect of PMP treatment described above, we further investigated whether the alleviation of hypoxic injury by PMP is related to its antioxidant regulation. The results showed that, compared with the model group, 0.1, 0.5 and 1.0 mg∙mL^−1^ PMP treatment significantly reduced ROS levels (*p* < 0.001, <0.001 and 0.008) (Figure 2A) and lipofuscin accumulation (*p* = 0.019, 0.001 and <0.001) (Figure 2B) in hypoxic *C. elegans*, effectively alleviating hypoxia-induced oxidative damage. Concurrently, 1.0 mg∙mL^−1^ PMP intervention significantly increased the survival rate of hypoxic *C. elegans* under oxidative stress (Figure 2C) and heat stress (Figure 2D), thereby enhancing their resistance to environmental stress. In summary, PMP treatment alleviates hypoxia-induced oxidative stress injury and enhances the stress resistance of hypoxic *C. elegans*. Furthermore, lifespan analysis results (Figure 2E) showed that *C. elegans* in the PMP group exhibited a certain extension trend compared with the model group. Although it did not reach statistical significance, it suggested that PMP treatment has a potentially beneficial effect on the lifespan of hypoxic *C. elegans*.

### 3.3. Protective Effect of PMP Intervention in Mice Exposed to Terminal Normobaric Hypoxia 

**Figure 3:**
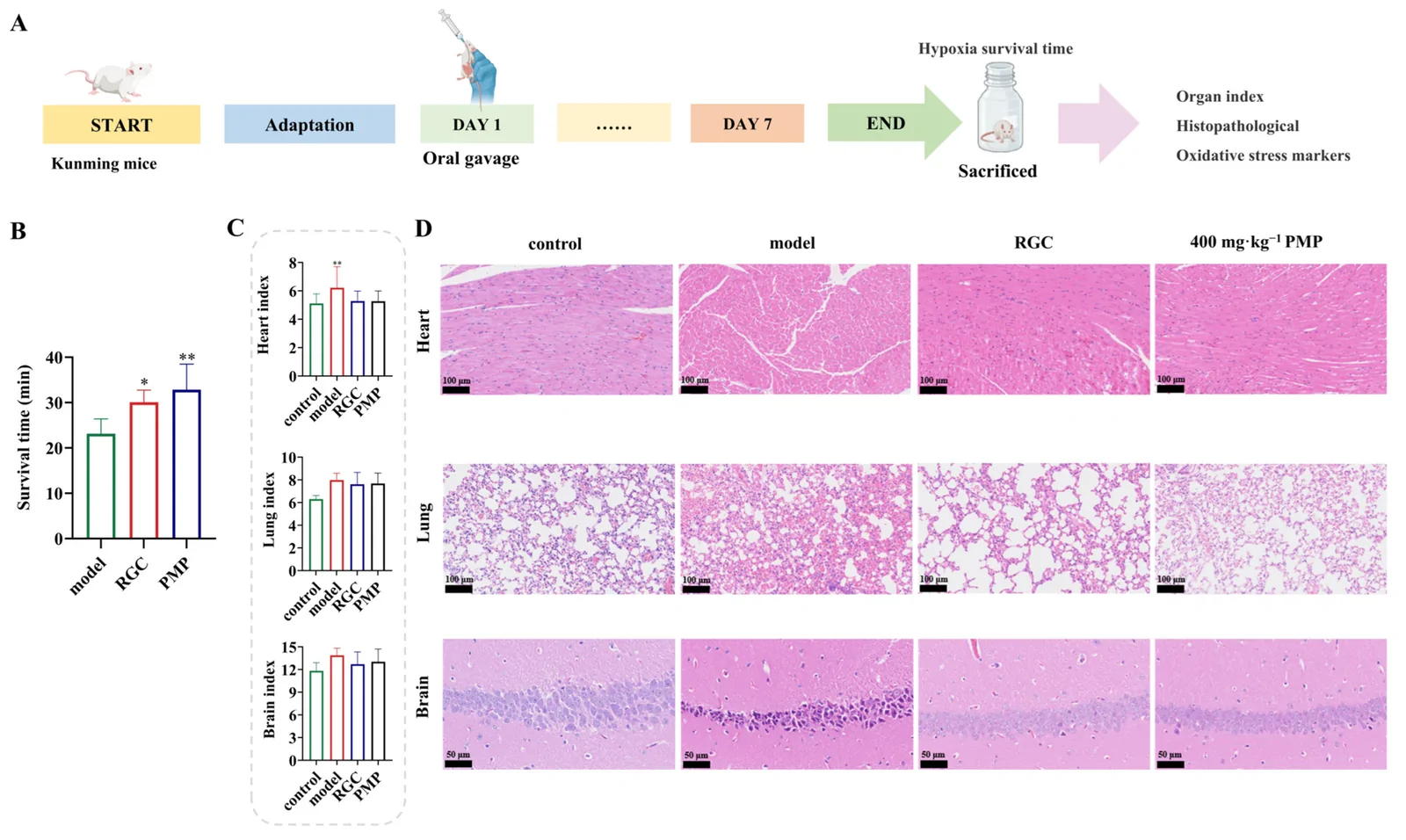
The protective effect of PMP intervention in mice exposed to terminal normobaric hypoxia. (**A**) Schematic illustration of the establishment of the terminal normobaric hypoxia mice model. Effects of PMP treatment on survival time (**B**), organ index (**C**) and heart (magnification 200×), lung (magnification 200×), and brain (magnification 400×) tissue pathology (**D**) in mice exposed to terminal normobaric hypoxia. (Note: Compared with the control group, * *p* < 0.05, ** *p* < 0.01. Control: normoxia + physiological saline; Model: hypoxia + physiological saline; RGC: hypoxia + RGC; PMP: hypoxia + PMP. The control group was normoxic control and did not participate in the comparison of survival time).

To investigate whether this protective effect is conserved in mammals, the terminal normobaric hypoxia mice model, which is more similar to humans in terms physiological characteristics, was further used for verification (Figure 3A). Compared with the model group (23.14 ± 3.28 min), 400 mg∙kg^−1^ PMP treatment significantly prolonged the survival time of hypoxic mice (32.86 ± 5.61 min), with a survival time extension rate of 42.01% (Figure 3B, Table S1). Brain, lung, and heart index decreased (Figure 3C, Table S2). This suggests that PMP intervention could also regulate organ function and state to a certain extent, alleviating hypoxia-induced organ damage. The heart, lung, and brain, as the main high oxygen-consuming organs of the body, are highly sensitive to hypoxic stimulation. Histopathological results showed that compared with the hypoxia model group, PMP intervention effectively improved hypoxia-induced organ pathological damage (Figure 3D). PMP exhibits a clear protective effect on hypoxia stress and injury at the whole-animal level.

### 3.4. Effects of PMP Intervention on the Body’s Stress Resistance Through Oxidative Stress Regulation in Mouse Model

**Figure 4:**
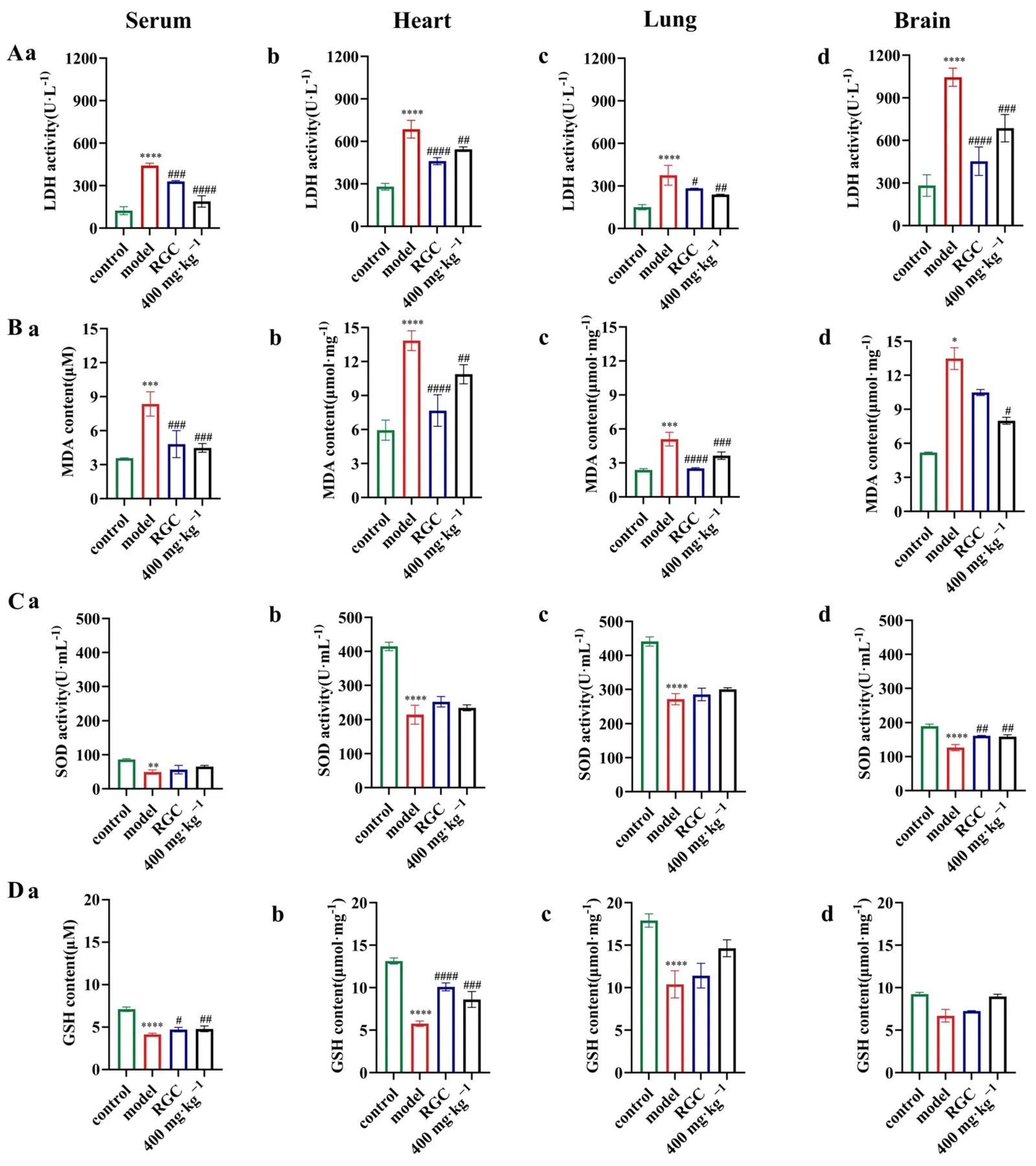
The verification of PMP enhancing the body’s stress resistance through oxidative stress regulation in mouse models. Effects of PMP treatment on oxidative stress markers in serum (**a**), heart (**b**), lung (**c**), and brain (**d**) tissues of mice exposed to terminal normobaric hypoxia. (**A**) LDH; (**B**) GSH; (**C**) MDA; (**D**) SOD. (Note: Control: normoxia + physiological saline; Model: hypoxia + physiological saline; RGC: hypoxia + RGC; PMP: hypoxia + PMP. Compared with control group, * *p* < 0.05, ** *p* < 0.01, *** *p* < 0.001, **** *p* < 0.0001; compared with model group, ^#^ *p* < 0.05, ^##^ *p* < 0.01, ^###^ *p* < 0.001, ^####^ *p* < 0.0001).

To further verify the conservation of anti-hypoxic effects of PMP dependent on oxidative stress regulation at the mammalian level, we measured the levels of oxidative stress and injury markers in mouse serum and in the heart, lung, and brain tissues. The results showed that PMP treatment significantly reversed the abnormally altered LDH activity (Figure 4A) and MDA concentration (Figure 4B), and restored SOD activity (Figure 4C) and GSH levels in hypoxic mice (Figure 4D). The decrease in LDH activity reflects the remission of cell membrane damage and metabolic disorders [24]. The reduction in MDA concentration indicates suppression of lipid peroxidation. The recovery of SOD activity and GSH levels indicates the functional reconstruction of the body’s enzymatic and non-enzymatic antioxidant defense system [25]. The systematic reversal of these indicators shows that PMP intervention effectively restores the body’s redox balance, improves cellular energy metabolism disorders, and then reduces the systemic physiological damage caused by hypoxia. These results are consistent with the ROS scavenging, lipofuscin reduction, and increased stress tolerance observed in *C. elegans*.

### 3.5. Effects of PMP Intervention on Endogenous Metabolism in C. elegans

**Figure 5:**
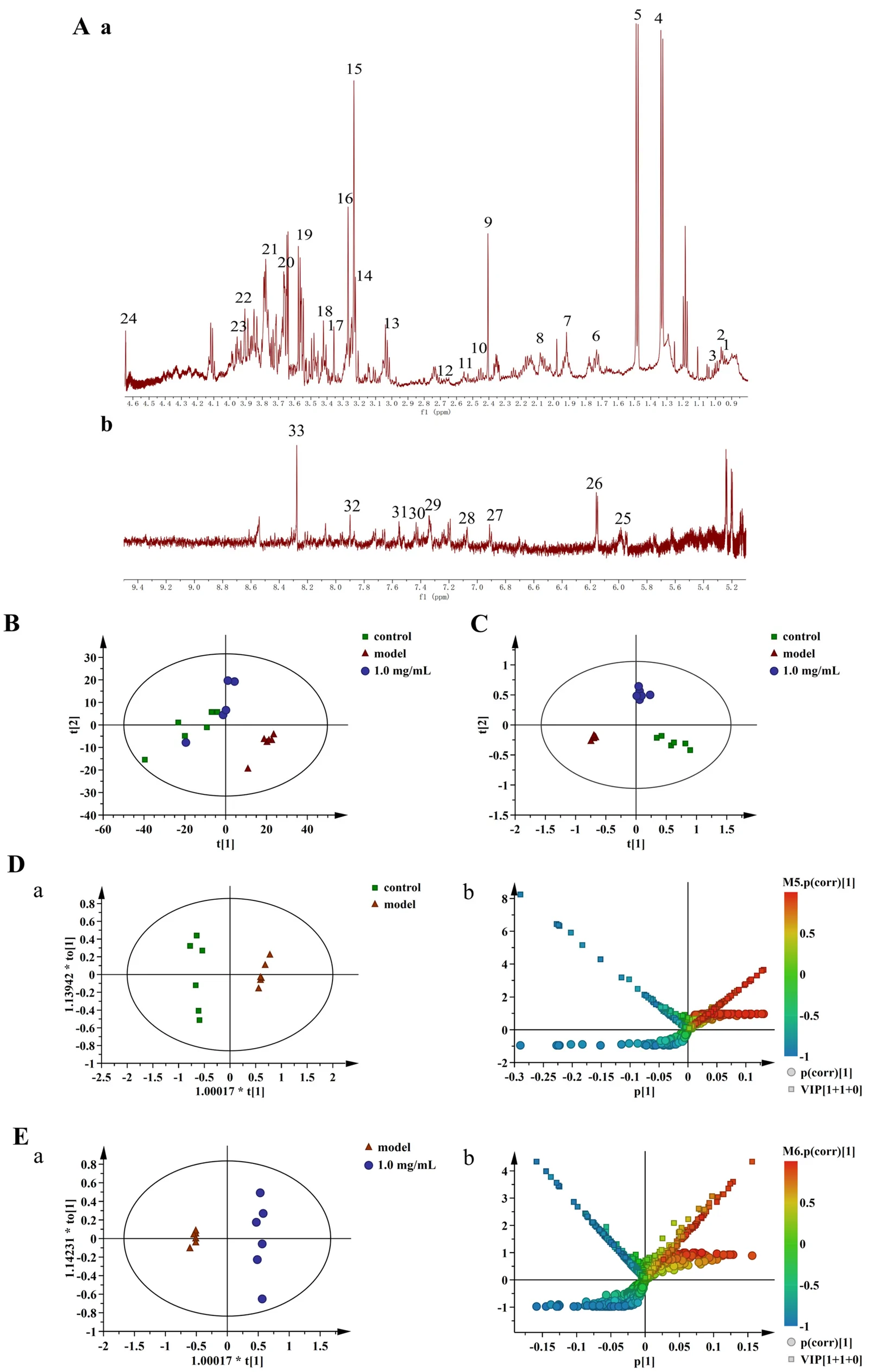
Effects of PMP intervention on endogenous metabolism in *C. elegans*. (**A**) Metabolite identification map of *C. elegans* endogenous metabolites: (**a**) low-intensity metabolite peak annotation; (**b**) high-intensity metabolite peak annotation. (**B**) PCA score plot. (**C**) PLS-DA score plot. (**D**) OPLS-DA score plot: (**a**) OPLS-DA analysis of blank and model groups; (**b**) S+V-plot score plot of blank and model groups. (**E**) OPLS-DA score plot: (**a**) OPLS-DA analysis of model group versus 1.0 mg·mL^−1^ PMP group; (**b**) S+V-plot score plot of model group versus 1.0 mg·mL^−1^ PMP group. (Note: Negative values on the coordinate axis are represented by hyphens, which represent negative numbers).

The improvement of PMP treatment on terminal markers of oxidative damage (ROS, GSH, etc.) caused by hypoxia suggests that its protective effect may not only be limited to passive clearance after injury, but also involve active regulation of upstream metabolic networks. ^1^H-NMR metabolomics was employed to systematically analyze changes in endogenous metabolites in hypoxic *C. elegans*. Thirty-three endogenous metabolites of *C. elegans* were identified from the ^1^H-NMR spectra (Figure 5A, Table S3). The PCA score plot showed that there was a certain separation trend between the control group, the model group, and the 1.0 mg·mL^−1^ PMP group (Figure 5B). T2 Range (Figure S3) and Dmodx (Figure S4) analyses were performed, and the results indicated that all samples should be retained. PLS-DA analysis revealed that the control group and the model group were significantly separated along the t [1] axis, and the 1.0 mg·mL^−1^ PMP group was closer to the control group (Figure 5C). The results of 200 permutation tests showed that the R^2^ intercept value was 0.33 (<0.4), and the Q^2^ intercept value was −0.40 (<0.0). The model had no over-fitting, indicating that the model was stable and effective (Figure S5). Further OPLS-DA analysis confirmed that the model group and the control group (R^2^X: 0.815, R^2^Y: 0.987, Q^2^: 0.968), and the model group and the PMP group (R^2^X: 0.706, R^2^Y: 0.995, Q^2^: 0.982) could be significantly distinguished (Figure 5D,E), indicating that PMP intervention had a significant overall corrective effect on hypoxia-induced metabolic disorders.

**Figure 6:**
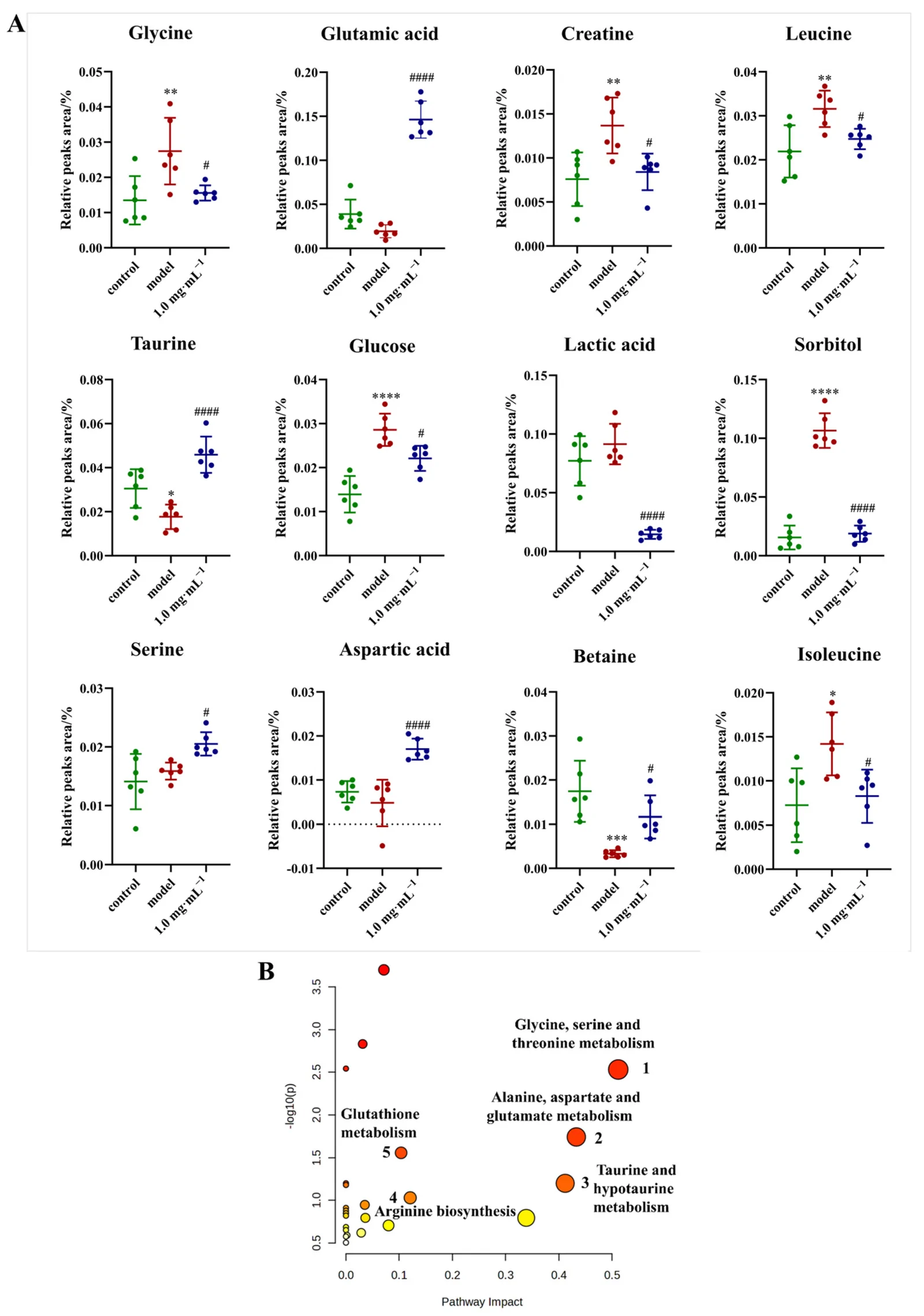
Effects of PMP intervention on endogenous metabolism in *C. elegans*. (**A**) Differentially regulated metabolites. (**B**) Topological map of related metabolic pathways (Note: Compared with the control group, * *p* < 0.05, ** *p* < 0.01, *** *p* < 0.001, **** *p* < 0.0001; compared with the model group, ^#^ *p* < 0.05, ^####^ *p* < 0.0001.)

Combined with S-plot score plot, VIP values, and independent samples t-test, a total of 12 significantly altered differential metabolites were identified (Figure 6A). Compared with the control group, the model group of hypoxic *C. elegans* exhibited significantly increased levels of glycine (*p* = 0.0082), creatine (*p* = 0.0050), leucine (*p* = 0.0045), glucose (*p* < 0.0001), lactic acid (*p* = 0.2913), sorbitol (*p* < 0.0001), serine (*p* = 0.5839), and isoleucine (*p* = 0.0121), while levels of glutamic acid (*p* = 0.1201), taurine (*p* = 0.0278), aspartic acid (*p* = 0.4664), and betaine (*p* = 0.0004) were significantly reduced. After PMP intervention, the levels of the above 12 metabolites were significantly reversed, confirming that PMP treatment can improve hypoxia-induced oxidative stress-related metabolic abnormalities by reshaping metabolic homeostasis. The differential metabolites were imported into Metaboanalyst 6.0 for analysis. According to the principle that the *p*-value < 0.1 and the impact factor > 0.1, five metabolic pathways were identified, which were glycine, serine and threonine metabolism; alanine, aspartate and glutamate metabolism; taurine and hypotaurine metabolism; and arginine biosynthesis and glutathione metabolism (Figure 6B and Figure S6). The involvement of multiple amino acid metabolic pathways, coupled with the significant downregulation of glucose and lactate levels after PMP intervention, mutually support the hypothesis that it alleviated hypoxia injury by maintaining cell energy metabolism. Furthermore, the regulation of key metabolites in taurine and glutathione metabolic pathways is also confirmed by the above phenotypic results regarding ROS, lipofuscin, and stress tolerance.

### 3.6. PMP Inhibits IIS Pathway and Activates DAF-16/FOXO to Mediate the Protective Effect Against Hypoxia Stress

**Figure 7:**
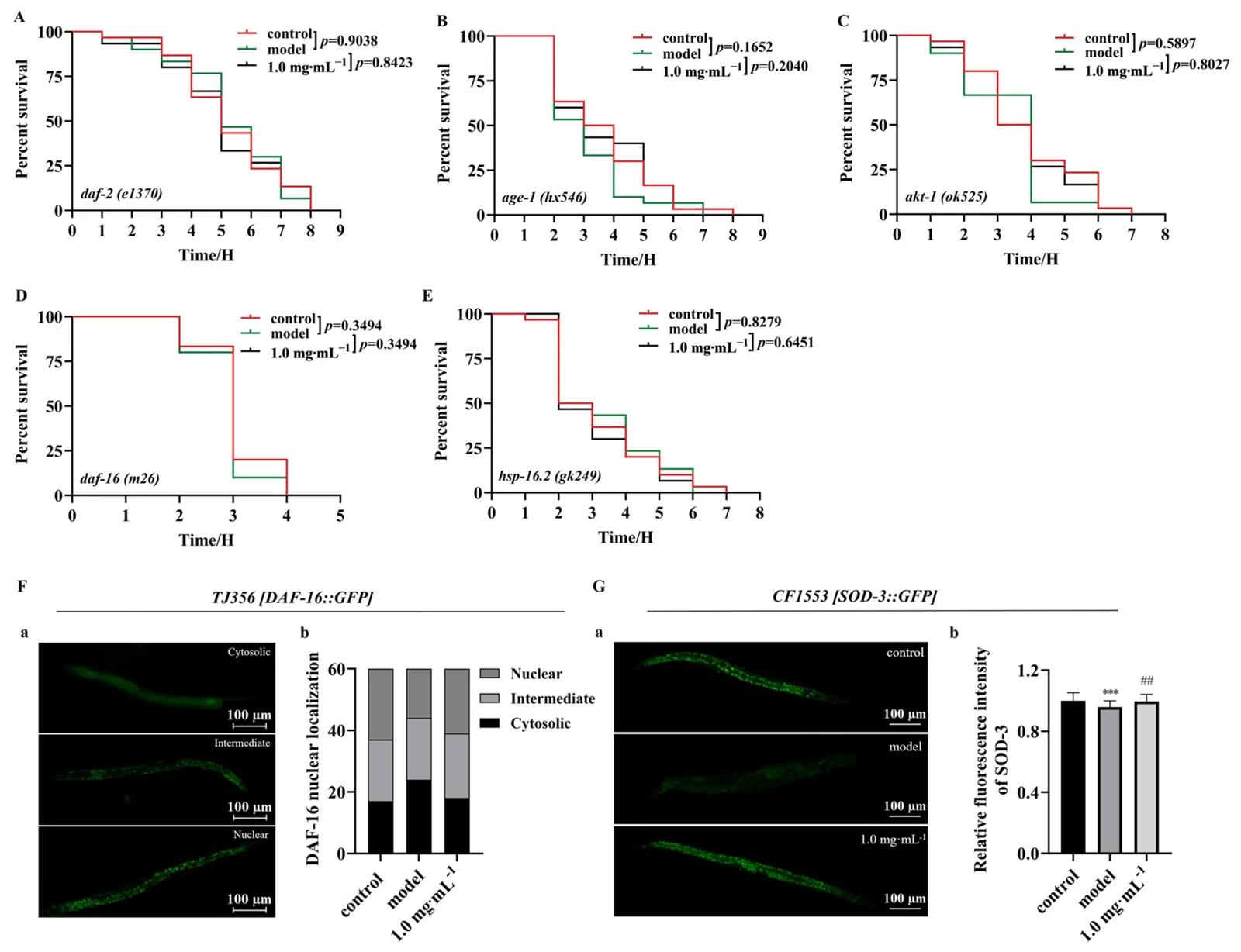
PMP inhibits IIS pathway and activates DAF-16/FOXO to mediate the protective effect against hypoxia stress. Effect of PMP on oxidative stress survival rate of *daf-2* (*n* = 120) (**A**), *age-1* (n = 120) (**B**), *akt-1* (*n* = 120) (**C**), *daf-16* (*n* = 120) (**D**), *hsp-16.2* (*n* = 120) (**E**) mutant *C. elegans* under hypoxia. (**F**) (**a**): Three expression forms of DAF-16 in *C. elegans*; (**b**): the effect of PMP treatment on the nuclear translocation ratio of *daf-16::GFP C. elegans* (*n* = 100). (**G**) (**a**): Representative pictures of *sod-3::GFP C. elegans* induced by PMP treatment; (**b**): the effect of PMP treatment on the relative fluorescence intensity of *sod-3::GFP C. elegans* (*n* = 100) (Note: Compared with the control group, *** *p* < 0.001; compared with the model group, ^##^ *p* < 0.01.)

The IIS pathway is an evolutionarily conserved hub for nutrient sensing and stress regulation. Inhibition of its activity activates the antioxidant defense system through DAF-16/FOXO to enhance stress tolerance [26], which is highly consistent with the effects of PMP verified in this study. We selected *C. elegans* with gene deficiencies at key nodes of the IIS pathway, including the insulin/IGF-1 receptor homolog *daf-2* (CB1370), the PI3K homolog *age-1* (TJ1052), the AKT kinase homolog *akt-1* (RB759), the FOXO transcription factor *daf-16* (DR26), and its downstream target gene *hsp-16.2* (VC475), to investigate the dependence of PMP anti-hypoxia protective effect on these genes. The results showed that the protective effect of PMP on hypoxic *C. elegans* under acute oxidative stress was eliminated in the above five mutants (Figure 7). Immunofluorescence and protein quantification results further showed that PMP intervention significantly promoted the nuclear translocation of DAF-16 (Figure 7F). SOD-3 is one of the core antioxidant target genes directly transcriptionally activated by DAF-16 upon nuclear entry. Consistent with this, PMP treatment significantly upregulated the expression level of SOD-3 protein in hypoxic *C. elegans* (Figure 7G). PMP treatment activates the downstream core transcription factor DAF-16/FOXO by inhibiting the IIS pathway.

### 3.7. DAF-16/FOXO Is a Key Node in PMP’s Regulation of Metabolic Reprogramming and Oxidative Stress in Hypoxic C. elegans

**Figure 8:**
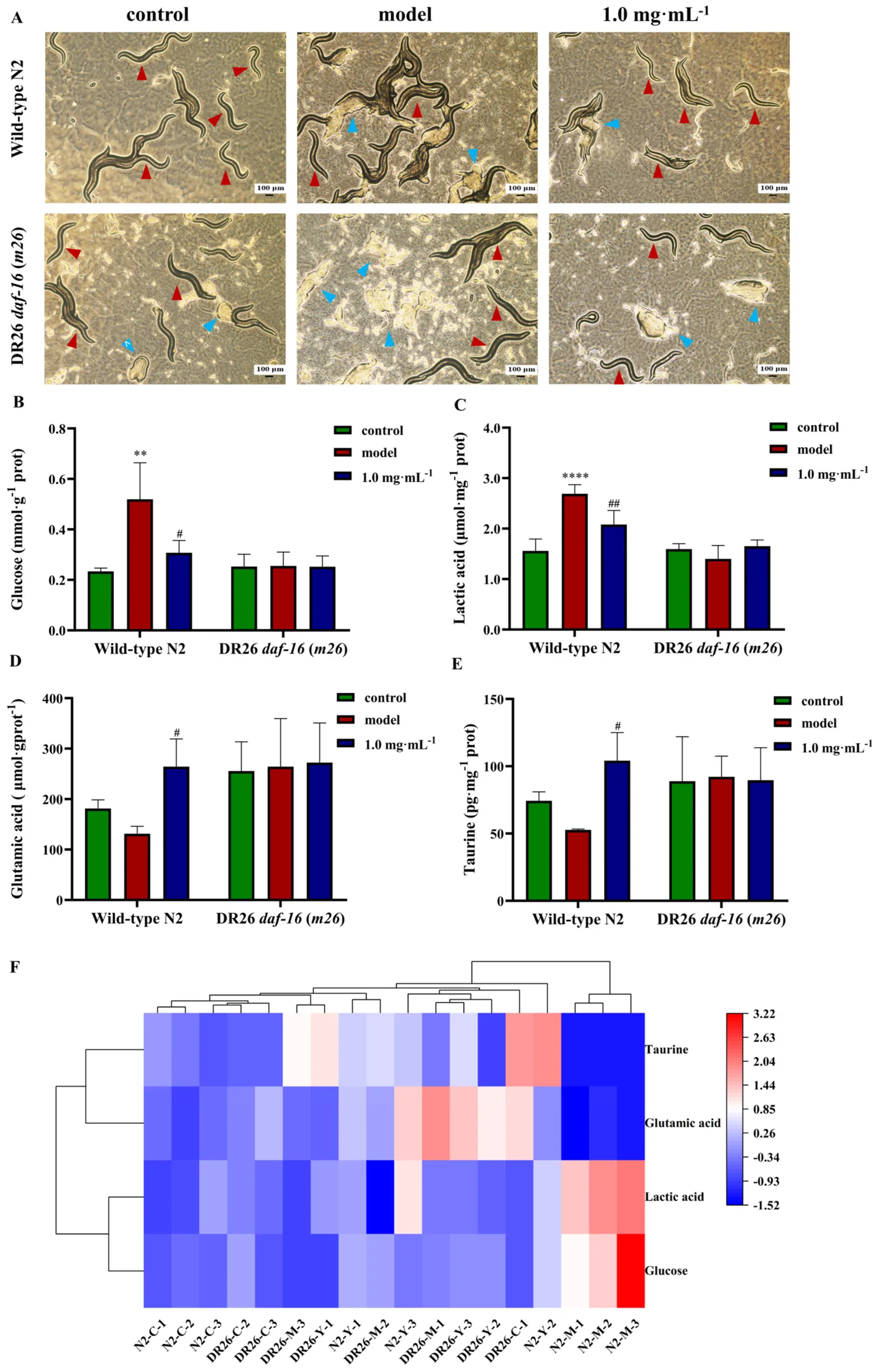
DAF-16/FOXO is a key node in PMP’s regulation of metabolic reprogramming and oxidative stress in hypoxic *C. elegans*. (**A**) The survival diagram of *C. elegans* 24 h after hypoxia-reoxygenation (survival: curved, red triangle; death: stiff, blue triangle). Effect of PMP treatment on the glucose (**B**), lactic acid (**C**), glutamic acid (**D**) and taurine (**E**) level in wild-type N2 and DR26 *daf-16* (*m26*) (Note: Compared with control group, ** *p* < 0.01, **** *p* < 0.0001; compared with model group, ^#^ *p* < 0.05, ^##^ *p* < 0.01.) (**F**) Effect of PMP treatment on the content of differential metabolites induced by hypoxia (Note: C: control, M: model, Y: 1.0 mg·mL^−1^ PMP. Negative values on the coordinate axis are represented by hyphens, which represent negative numbers).

To verify whether DAF-16/FOXO, the downstream transcription factor of the IIS pathway, mediates the regulatory effects of PMP on metabolism and oxidative stress in hypoxic *C. elegans*, we first compared the survival status of N2 and *daf-16*-deficient *C. elegans* after hypoxia-reoxygenation for 24 h. The results showed that the survival status of N2 *C. elegans* was better than that of *daf-16*-deficient *C. elegans* (Figure 8A). This indicates that DAF-16 is an essential factor required for *C. elegans* to resist hypoxia injury. Based on the 12 differential metabolites and four metabolic pathways identified in the previous metabolomics analysis, we selected glucose, lactic acid, glutamic acid, and taurine as representatives to examine their content changes after hypoxia and PMP intervention in N2 and *daf-16*-deficient *C. elegans*, respectively. In N2 *C. elegans*, hypoxia stress led to the accumulation of glucose (*p* = 0.0011) (Figure 8B) and lactic acid (*p* = 0.0008) (Figure 8C), and the consumption of glutamic acid (*p* = 0.5865) (Figure 8D) and taurine (*p* = 0.4092) (Figure 8E). PMP intervention significantly reversed the above changes, which were consistent with the previous metabolomics results. In *daf-16*-deficient *C. elegans*, there was no significant difference in the content of the above four metabolites among the groups (Figure 8F). DAF-16 deficiency blocked the regulation of PMP intervention on hypoxia-related metabolism. It can be seen that DAF-16/FOXO, as the core transcription factor of the IIS pathway, is an essential upstream regulatory node for PMP-mediated energy metabolism remodeling and antioxidant homeostasis maintenance in hypoxic *C. elegans*.

### 3.8. Material Basis of Anti-Hypoxia Activity of PMP and Screening of Core Active Peptides

**Figure 9:**
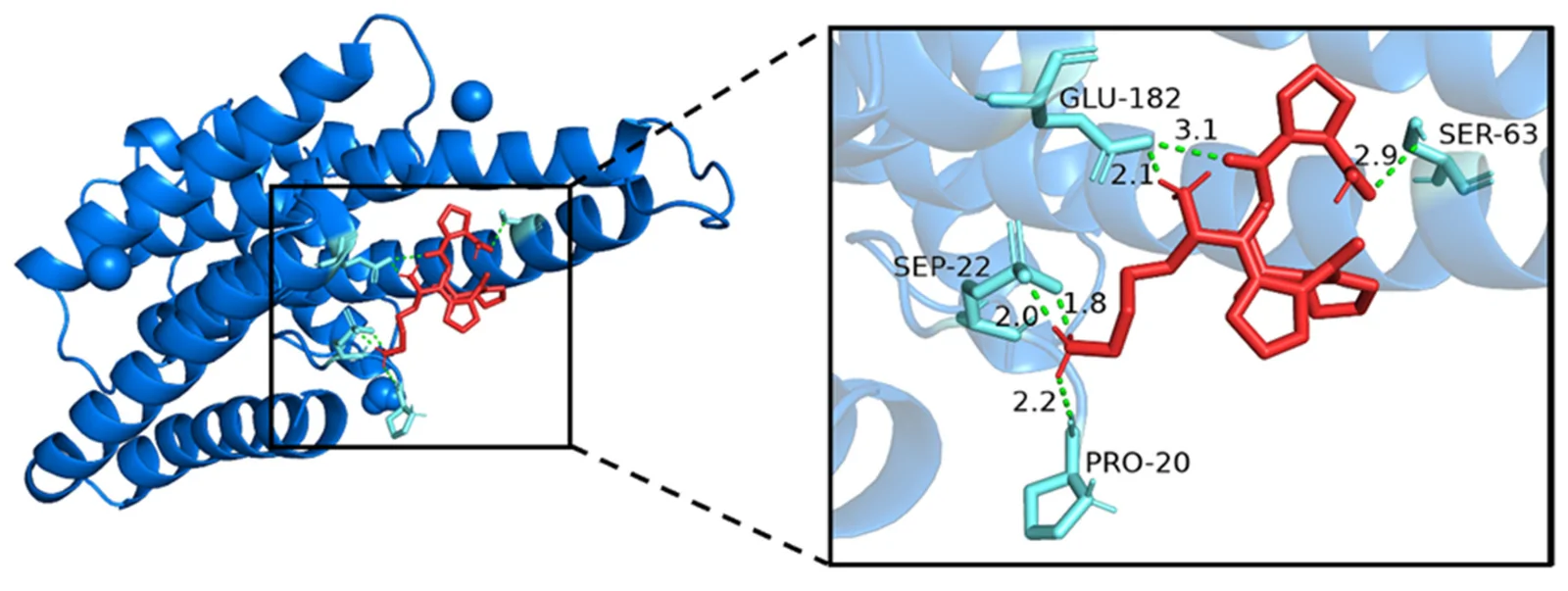
Molecular docking of PMP and FOXO1 protein: KPPGP formed six hydrogen bonds with GLU-182, SER-63, SEP-22 and PRO-20 and is embedded within FOXO1.

Treatment with PMP imparted good resistance to hypoxia injury in both *C. elegans* and mouse models. Next, we explored the material basis of PMP. The total ion flow diagram of PMP is shown in Figure S7. A total of 130 peptides were identified in the positive ion mode by Peaks de Novo sequencing analysis, and the average local confidence of the peptides was higher than 50% (Table S4). The representative peptide VLSAADKAVNK was selected to verify the accuracy of the key peptide sequence, and its MS/MS spectrum showed highly reliable fragment ion matching (Figure S8). Using PeptideRanker, the scoring threshold (≥0.80) was set to predict the biological activity of each peptide, and a total of 26 high-scoring peptides were obtained. Through BIOPEP-UWM prediction, 26 peptide sequences were input one by one to analyze whether they contained biologically active amino acid residues related to antioxidants. The results showed that a total of 12 peptide segments contained antioxidant active amino acid residues. Further analysis of the active sites within these peptide segments, including GC, GP, VC, etc., showed that these repeated short peptide sequences may be the key active sites of antioxidant function and thus hold potential for further development (Table 1). The human homologue FOXO1 of the key target DAF-16 protein on the IIS pathway was selected to perform molecular docking simulation on each of the 12 peptides containing potential antioxidant motifs (Table 1). Among them, the binding energy of KPPGP was -4.5 Kcal/mol, and six hydrogen bonds were formed with GLU-182, SER-63, SEP-22, and PRO-20 embedded in FOXO1 (Figure 9). In order to further evaluate the drug potential of peptides, the physicochemical properties, biosafety, toxicity, and intestinal absorption characteristics of 12 peptides were systematically analyzed using various biological tools (Table 1). Among them, KPPGP had better water solubility, and the other 11 peptides had poor water solubility. The stability of the peptides was evaluated by Expasy, and six peptides were stable and had a long action time in vivo. Further toxicity was predicted by ToxinPred, and KPPGP and AWCC were predicted to be non-toxic, with good biosafety and suitable as potential therapeutic molecules. Subsequently, the intestinal absorption capacity of the peptides was predicted using ADmet SAR. With the exception of VCCPGCC, GVCPCCC, CCVGPCC, and PVCGCCC, the remaining eight peptides tested positive for HIA, indicating favorable intestinal absorption properties. Peptide identification is currently based on high-resolution mass spectrometry and bioinformatics analysis; validation using synthetic peptides will be the focus of our subsequent research.

## 4. Discussion

Hypoxia triggers mitochondrial dysfunction in *C. elegans* [27,28], activates the antioxidant enzyme system, and causes an increase in ROS levels. Persistent hypoxia leads to lipid peroxidation damage. Free radicals attack polyunsaturated fatty acids in membrane phospholipids, triggering chain peroxidation, generating MDA, 4-hydroxynonenal, and other products that cross-link with proteins. The damaged proteins combine with lipid peroxides and promote lipofuscin deposition [29], ultimately causing cell death and tissue injury. In the *C. elegans* model, PMP treatment significantly reduced the levels of ROS and lipofuscin in hypoxic *C. elegans* and improved their survival under oxidative stress. In the terminal normobaric hypoxia mice model, it enhanced SOD activity, elevated GSH levels, and reduced MDA concentrations. These results indicate that one of the core effects of PMP treatment is the restoration of redox homeostasis. However, oxidative stress is not an isolated event. Excessive ROS directly inhibits mitochondrial respiratory chain complex I/III, reduces ATP synthesis efficiency [30], and leads to a decrease in NAD ^+^/NADH ratio, which in turn feeds back to enhance glycolytic flux [31]. LDH is a key enzyme in glycolysis, and its elevated serum levels typically reflect increased cell membrane permeability and metabolic disorders [32]. MDA is an end product of lipid peroxidation, and lipid metabolism is closely related to energy metabolism. At the same time, metabolic intermediates, such as NADPH and pyruvate, regulate cellular antioxidant capacity through ways such as reduced GSH regeneration. The recovery of the antioxidant system after treatment could reduce the inhibition of ROS on the mitochondrial electron transport chain, thereby improving energy production efficiency [33]. In short, hypoxia injury is accompanied by a vicious cycle of redox imbalance and metabolic disorders. PMP shows consistent antioxidant effects in both *C. elegans* and mice, suggesting that the molecular mechanism underlying its action is conserved across species. Previous studies have shown that many regulatory mechanisms in the hypoxia response of *C. elegans* are conserved in mammals, highlighting the unique value of *C. elegans* in hypoxia mechanism research [34]. Based on this conservation and the overall biological advantages and high genetic operability of *C. elegans*, it was selected as the model to further elucidate the mechanism of PMP against hypoxia.

When oxidative phosphorylation is impaired, cells are forced to turn to glycolysis for extra energy supply, but this compensation is often insufficient, resulting in abnormal accumulation of metabolic intermediates [30]. In fact, the impact of hypoxia on the metabolic network is far beyond energy metabolism itself, involving systemic changes in multiple pathways such as amino acid metabolism and antioxidant metabolism [35]. A total of 12 differential metabolites were identified by ^1^H-NMR metabolomics in hypoxic *C. elegans*. MetaboAnalyst enrichment analysis identified five metabolic pathways: glycine, serine and threonine metabolism; alanine, aspartic acid and glutamic acid metabolism; taurine and hypotaurine metabolism; and arginine biosynthesis and glutathione metabolism. Alanine, aspartate and glutamate metabolism are closely related to energy metabolism. Hypoxia inhibits mitochondrial respiration, but simultaneously enhances glycolysis to compensate for energy metabolism [36]. However, ATP produced by glycolysis is not enough to compensate for demand, resulting in metabolic obstruction and accumulation of metabolic intermediates [37]. Glycine, serine and threonine metabolism, taurine metabolism, and glutathione metabolism are directly related to the antioxidant defense system, indicating that hypoxia was able to induce systemic metabolic disorders across multiple pathways, such as amino acid metabolism and antioxidant metabolism. GSH would protect protein sulfhydryl groups and regulate redox-sensitive signaling pathways to enhance cellular antioxidant capacity [38]. Its biosynthesis depends on glutamate, cysteine, and glycine [39]. The regulation of glutamate metabolism and glycine and serine metabolism by PMP treatment directly reflects the synthesis status of GSH. Alanine, aspartic acid, and glutamic acid metabolism are the hubs connecting glycolysis and the tricarboxylic acid cycle [40]. The regulation of these pathways by PMP intervention may optimize carbon flux, help cells utilize limited oxygen to generate energy more efficiently under hypoxic conditions, and maintain energy homeostasis. This improvement may be related to the decrease in LDH level. Taurine has been shown to have significant antioxidant, anti-apoptotic, and neuroprotective properties, which can stabilize cell membranes and calcium homeostasis. Its consumption reflects the depletion of antioxidant reserves in the body [41]. Arginine is not only a substrate for protein synthesis, but also a precursor of NO, which is involved in vasodilation and neurotransmission [42,43]. This study showed that PMP treatment could enhance glutathione metabolism, coordinate energy metabolism, and activate amino acid metabolism to enhance the body’s antioxidant capacity. Ultimately, through the synergistic effect of multiple targets and multiple pathways, PMP can effectively repair oxidative damage and energy disorders caused by hypoxia.

In the field of antioxidation and anti-hypoxia, various natural products have been reported to exert their effects, and there are multiple pathways involved. Nrf2 is one of the most classic antioxidant regulatory hubs. Recent studies have reported that bioactive peptides isolated from yak milk protect against hypoxia-induced renal injury and lung injury through the Nrf2 pathway [44]. The IIS pathway is the core pathway regulating stress resistance, metabolism, and lifespan in cells [26,45]. When the IIS pathway is inhibited (e.g., under stress conditions), reduced AKT-1 activity leads to DAF-16 dephosphorylation and nuclear translocation, which binds to the DBE in the promoter region of the target gene. These regulations activate the antioxidant defense system (*sod-3*, *ctl-1*), molecular chaperones (*hsp-16.2*), autophagy-related genes (*lgg-1*), and metabolic regulatory genes, while simultaneously inhibiting lipid synthesis and enhancing gluconeogenesis [46]. This dynamic regulatory network integrates external nutrient signals with cellular stress responses, and ultimately affects the lifespan and stress resistance of *C. elegans*. PMP treatment induced nuclear translocation of DAF-16 and upregulated the expression of its downstream target gene SOD-3, suggesting that PMP activates this key transcription factor by inhibiting upstream signals. More importantly, in the *daf-16* deletion mutant, PMP treatment not only failed to prolong the survival time, but also completely lost its ability to reverse metabolic disorders. This demonstrates that DAF-16 is a necessary node for PMP to exert anti-hypoxia protection. It has been reported that the FOXO transcription factor serves as a signal integrator to maintain homeostasis, playing a protective role by regulating cell cycle, apoptosis, oxidative stress resistance, and metabolism. FOXO is activated by metabolic stress and oxidative stress [47]. Another study further confirmed that the FOXO/DAF-16 regulatory gene expression network is widely involved in energy production and cell stress responses [48]. Our experimental data are highly consistent with this theory and provide new experimental evidence that the IIS/FOXO pathway serves as a key molecular node connecting oxidative stress and energy metabolism under hypoxic conditions. Notably, reduced IIS pathway activity prolonged lifespan in various model organisms in mammals, suggesting that targeting this pathway may have broader therapeutic application prospects [49].

This study was the first to demonstrate that bioactive peptides derived from *P*. *manillensis* had anti-hypoxic effect, and established a link between the IIS/FOXO pathway and the regulation of oxidative stress and metabolic disorders under hypoxia. In addition, the sequence identification and docking characteristics of KPPGP provide a reference for peptide research. However, the binding of KPPGP to FOXO1 has been demonstrated only through computational simulations. Whether it can independently activate the transcription or act as a ligand still needs verification by in vitro binding assays and bioactivity experiments. In the detection of metabolite levels in *C. elegans*, we only selected some representative metabolites for verification and were unable to fully cover all differential metabolites identified by non-targeted metabolomics, which limits the description of the metabolic reprogramming. In addition, although we observed improvements in biochemical indicators such as SOD and GSH in the terminal normobaric hypoxia mice model, the expression levels of FOXO downstream target genes in mice exposed to terminal normobaric hypoxia have not been directly measured. This verification would further strengthen the causal relationship between PMP and the IIS/FOXO pathway. Both the sodium sulfite chemical hypoxia model of *C. elegans* and the normobaric hypoxia model of mice used in this study were acute hypoxia exposures, and PMP’s efficacy in scenarios such as chronic or intermittent hypoxia, or ischemia-reperfusion, requires further validation in subsequent studies.

## 5. Conclusions

In summary, in *C. elegans*, PMP inhibits the IIS pathway and activates the downstream transcription factor DAF-16/FOXO, thereby coordinating metabolic reprogramming and antioxidant defense to resist hypoxia-induced oxidative damage and metabolic disorders; this protective effect was also observed in the mouse model. This finding reveals for the first time that the IIS/FOXO axis serves as a critical hub connecting oxidative stress and energy, amino acid, and other metabolic pathways under hypoxic conditions. Additionally, DAF-16 is an essential factor for PMP to exert anti-hypoxic effect and could be used as a potential new target for anti-hypoxic intervention. In addition, KPPGP identification also provides a hypothesis for the discovery and functional study of natural bioactive peptides.

## Figures and Tables

**Table 1:** Identification of physical and chemical properties of antioxidant active peptides.

No.	Peptide Sequence	Formula	Peptide Rank Score	Valid Sequence	Toxicity	Solubility	Instability	Instability (Mammalian Reticulocytes, In Vitro)	ADMET	Length	Mass
1	HDCCCCGCCCMCCHH	C_56_H_85_N_21_O_18_S_10_	0.992877	HH, GC	Toxin	Poor	52.8	3.5 h	HIA+	15	1659.36
2	AWCC	C_20_H_25_N_5_O_5_S_2_	0.981333	AW	Non-Toxin	Poor	/	/	HIA+	4	481.15
3	ACMPGCC	C_24_H_41_N_7_O_8_S_4_	0.967793	GC	Toxin	Poor	180.69	4.4 h	HIA+	7	683.19
4	ACMGPCC	C_24_H_41_N_7_O_8_S_4_	0.960273	GP	Toxin	Poor	107.14	4.4 h	HIA+	7	683.19
5	SGWCTCC	C_29_H_42_N_8_O_10_S_3_	0.957701	GW	Toxin	Poor	72.77	1.9 h	HIA+	7	758.22
6	VCCPGCC	C_24_H_41_N_7_O_8_S_4_	0.941219	GC, VC	Toxin	Poor	36.09	100 h	HIA−	7	683.19
7	GVCPCCC	C_24_H_41_N_7_O_8_S_4_	0.940204	VC	Toxin	Poor	25.31	30 h	HIA−	7	683.19
8	MPCQCC	C_24_H_41_N_7_O_8_S_4_	0.918813	CQC	Toxin	Poor	43.87	30 h	HIA+	6	683.19
9	CCVGPCC	C_24_H_41_N_7_O_8_S_4_	0.918431	GP	Toxin	Poor	−25.1	1.2 h	HIA−	7	683.19
10	PMCQCC	C_24_H_41_N_7_O_8_S_4_	0.910964	CQC	Toxin	Poor	−29.37	>20 h	HIA+	6	683.19
11	PVCGCCC	C_24_H_41_N_7_O_8_S_4_	0.904542	GC, VC	Toxin	Poor	36.09	>20 h	HIA−	7	683.19
12	KPPGP	C_23_H_38_N_6_O_6_	0.806035	KP, GP	Non-Toxin	Good	31.44	1.3 h	HIA+	5	494.29

## Data Availability

The raw data supporting the conclusions of this article will be made available by the authors on request.

## Supplementary Materials

The following supporting information can be downloaded at https://www.mdpi.com/article/10.3390/antiox15080936/s1. Figure S1: Establishment of the hypoxia model in *C. elegans*; Figure S2: Screening of non-toxic doses of PMP for *C. elegans*; Figure S3: T2 Range; Figure S4: Dmodx; Figure S5: The permutation test diagram; Figure S6: The metabolic pathways; Figure S7: Total ion current diagram of PMP; Figure S8: Peptide sequence analysis based on MS/MS spectra; Table S1: Effects of PMP on survival of hypoxic mice; Table S2: Effects of PMP on brain, lung and heart indexes of hypoxic mice; Table S3: Endogenous metabolite signal attribution; Table S4: Peptide signal attribution.

## Abbreviations

AKT-1	Serine/threonine kinase 1
ATP	Adenosine triphosphate
*C. elegans*	*Caenorhabditis elegans*
DAF-16	Abnormal dauer formation-16
DBE	Double bond equivalent
*E. coli* OP50	*Escherichia coli* OP50
FDR	False discovery rate
FOXO	Forkhead Box O
GSH	Glutathione
GSSG	Glutathione disulfide
HIA	Human intestinal absorption
HIF	Hypoxia-inducible factor
^1^H-MNR	Proton nuclear magnetic resonance
IIS	Insulin/insulin-like growth factor-1 signaling
LDH	Lactate dehydrogenase
MAPK	Mitogen-activated protein kinase
MDA	Malondialdehyde
NAD^+^	Nicotinamide adenine dinucleotide
NADH	Reduced nicotinamide adenine dinucleotide
NADPH	Reduced nicotinamide-adenine dinucleotide phosphate
NF-κB	Nuclear factor kappa-light-chain-enhancer of activated B cells
NGM	Nematode growth medium
Nrf2	Nuclear factor erythroid 2-related factor 2
OPLS-DA	Orthogonal partial least squares discriminant analysis
PCA	Principal component analysis
PI3K	Phosphoinositide 3-Kinase
PLS-DA	Partial least squares discriminant analysis
PMP	*Poecilobdella manillensis* bioactive peptide
RGC	Rhodiola Ginseng Capsules
RNAi	RNA interference
ROS	Reactive oxygen species
TFA	Trifluoroacetic acid
UPLC-Q-Exactive-MS	Ultra-performance liquid chromatography-Quadrupole-Exactive Orbitrap Mass Spectrometry
VIP	Variable importance in projection
SOD	Superoxide dismutase
SPE	Solid-phase extraction
