# Supplementary material for: Poecilobdella manillensis Bioactive Peptides Reduce Oxidative Stress and Regulate Metabolic Reprogramming via the IIS/FOXO Pathway to Improve Hypoxic Injury
Source: Antioxidants (Basel). 2026 Jul 28;15(8):936. doi: 10.3390/antiox15080936 (PMC13509369; doi:10.3390/antiox15080936)
Supplement: Supplementary file 1 [file antioxidants-15-00936-s001.zip › antioxidants-4368669-supplementary.pdf]

Poecilobdella manillensis bioactive peptides reduce oxidative stress and regulate metabolic reprogramming via the IIS/FOXO pathway to improve hypoxic injury

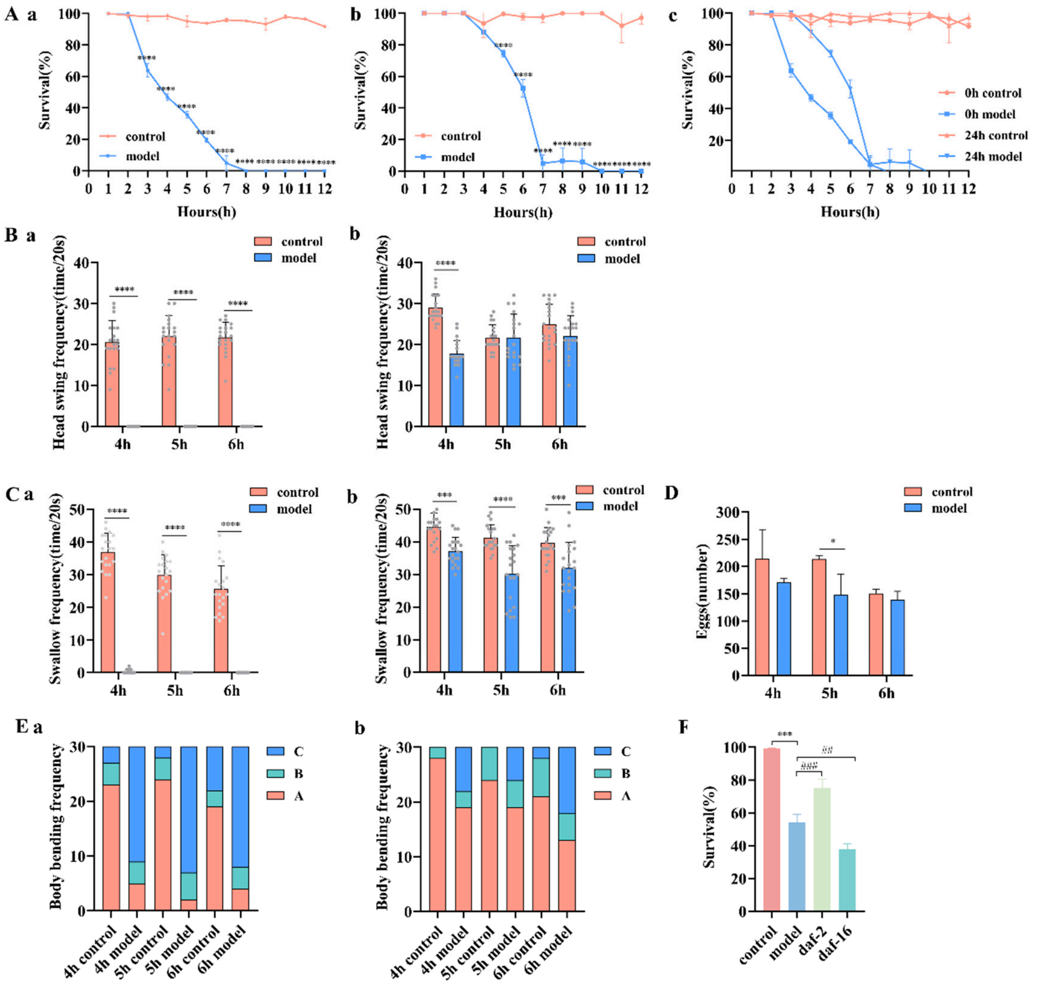

Fig. S1. Establishment of the hypoxia model in *C. elegans*: (A) Survival rates of *C. elegans* under different hypoxic durations, a. Reoxygenation 0 h, b. Reoxygenation 24 h, c. comparison chart between 0 h and 24 h. (B) Head swing frequency of *C. elegans* under different hypoxic durations (n=20), a. Reoxygenation 0 h, b. Reoxygenation 24 h. (C) Swallow frequency of *C. elegans* under different hypoxic durations (n=20), a. Reoxygenation 0 h, b. Reoxygenation 24 h. (D) Egg production of *C. elegans* under different hypoxic durations (n=10), a. Reoxygenation 0 h, b. Reoxygenation 24 h. (E) Body bending frequency of *C. elegans* under different hypoxic durations (n=30), a. Reoxygenation 0 h, b. Reoxygenation 24 h. (F) Survival rates of *C. elegans* from different strains after treatment with 2.0 g·L^{-}

[1] sodium sulfite for 6 h and reoxygenation for 24 h (Note: Compared with the control group, *$P < 0.05$, **$P < 0.01$, ***$P < 0.001$, ****$P < 0.0001$; compared with the model group, $^{\#}P < 0.05$, $^{\#\#}P < 0.01$, $^{\#\#\#}P < 0.001$, $^{\#\#\#\#}P < 0.0001$.) It is feasible and effective to establish a chemical hypoxia model for *C. elegans* using a 2.0 $g \cdot L^{-1}$ sodium sulfite solution for 6 h.

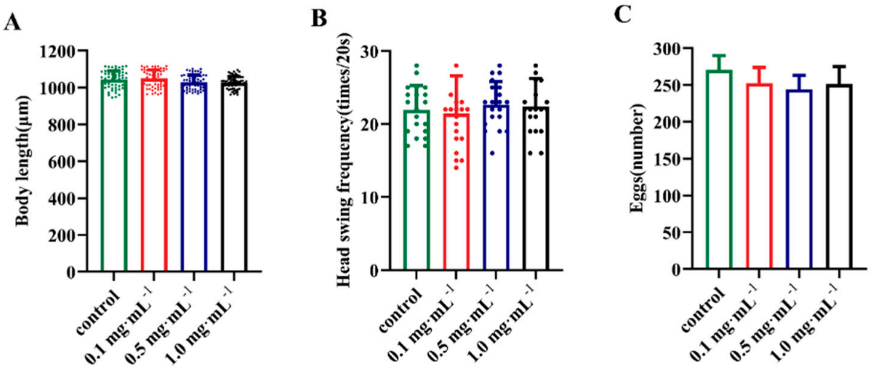

Fig. S2 Screening of non-toxic doses of PMP for *C. elegans*: (A) Effect of PMP on the body length of *C. elegans* (n=60). (B) Effect of PMP on the head swing frequency of *C. elegans* (n=20). (C) Effect of PMP on the egg production of *C. elegans* (n=10). At the concentrations of 0.1, 0.5 and 1.0 $mg \cdot mL^{-1}$, PMP had no effect on the growth and development, feeding and reproduction of *C. elegans*.

Table S1 Effects of PMP on survival of hypoxic mice（$\bar{x}$ ± s， n = 10）.

Groups	Dosage（mg/kg·d）	Survival time（min）	Prolonged rate of survival time （%）
control	/	/	/
model	/	23.14 ± 3.28	0.00
positive control	266.6	30.08 ± 2.69$^{\#}$	29.99
PMP	400.0	32.86 ± 5.61$^{\#\#}$	42.01

(1) Compared with the control group, *$P < 0.05$, **$P < 0.01$; (2) compared with the model group, $^{\#}P < 0.05$, $^{\#\#}P < 0.01$.

Table S2 Effects of PMP on brain, lung and heart indexes of hypoxic mice（$\bar{x}$ ± s， n = 10）.

Groups	Dosage (mg/kg·d)	Brain index	Lung index	Heart index
control	/	11.83 ± 1.07	6.30 ± 0.33	5.11 ± 0.67
model	/	13.87 ± 0.95	7.97 ± 0.60	6.22 ± 1.48**
positive control	266.6	12.70 ± 1.62	7.61 ± 1.05	5.28 ± 0.70
PMP	400.0	13.03 ± 1.68	7.68 ± 0.93	5.26 ± 0.73

(1) Compared with the control group, *$P < 0.05$, **$P < 0.01$; (2) compared with the model group, #$P < 0.05$, ##$P < 0.01$.

Table S3 Endogenous metabolite signal attribution.

NO.	Metabolite	Peak pattern	δ_H
1	Isoleucine	d; m	1.01; 0.93
2	Leucine	d; d	0.97; 0.96
3	valine	d; d	0.99; 1.05
4	Lactic acid	q; d	4.11; 1.33
5	Alanine	d	1.49
6	Lysine	m; m; q; m	1.73; 1.92; 3.03; 3.66
7	Acetic acid	s	1.92
8	Glutamic acid	m; m; m; m	3.77; 2.36; 2.12; 2.07
9	Succinic acid	s	2.41
10	Glutamine	m; m; m	3.77; 2.44; 2.14
11	Citric acid	d; d	2.70; 2.54
12	Aspartic acid	m; dd; m	3.90; 2.82; 2.69
13	Creatine	s; s	3.93; 3.04
14	Choline	s	3.22
15	Phosphatidylcholine	s	3.23
16	Trimethylamine oxidase	s	3.27
17	Taurine	t; t	3.42; 3.26
18	Glycine	s	3.56
19	Sorbitol	m; m	3.85; 3.66
20	2-Aminoglycolic acid	t	3.77
21	betaine	s	3.91
22	serine	dd; dd; dd	3.99; 3.95; 3.85
23	Glucose	d; d; m; m; t	5.24; 4.65; 3.69-3.93; 3.39-3.55; 3.25
24	Uridine diphosphate-N-acetylglucosamine	d; d; d; dd	7.96; 5.99; 5.97; 5.52
25	Inosine	m/m; s	6.15; 8.21; 8.34
26	Tyrosine	d; d	7.20; 6.90
27	Histidine	s; s; dd	7.84; 7.08; 3.99
28	Tryptophan	d; d; t; t; s; m; m; m	7.74; 7.54; 7.33; 7.29; 7.20; 4.05; 3.49; 3.32
29	Phenylalanine	m; m; m	7.43; 7.38; 7.33
30	Uracil	d; d	7.54 ; 5.80
31	Xanthine	s	7.91
32	Adenosine diphosphate	s; s; d; m	8.54; 8.27; 6.14; 4.60
33	Adenosine monophosphate	s; s; d	8.61; 8.27; 6.15

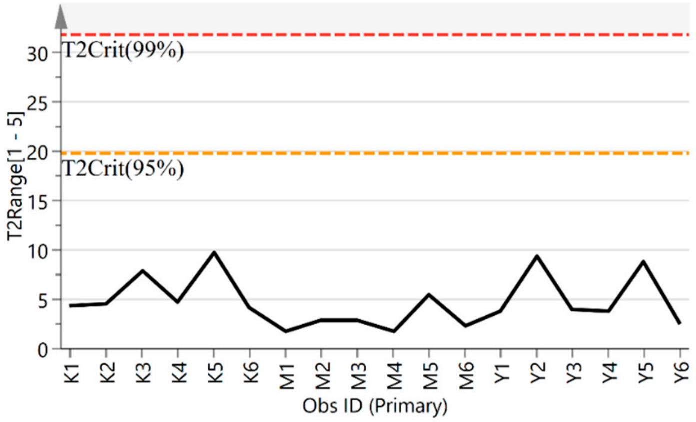

Fig. S3 T2 Range.

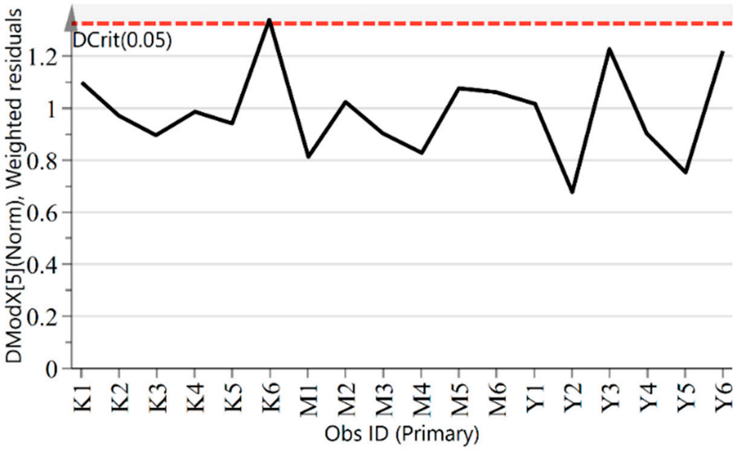

Fig. S4 Dmodx.

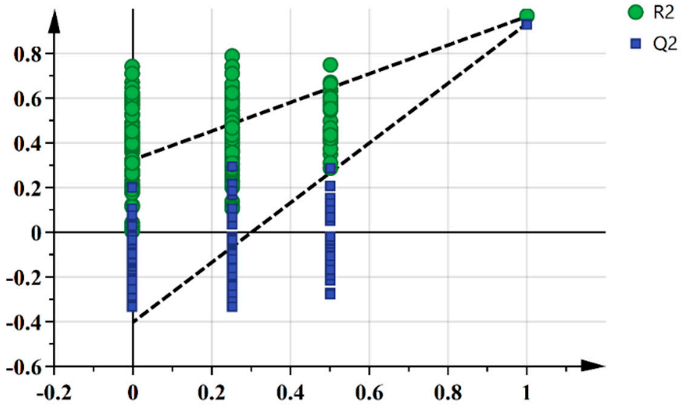

Fig. S5 The permutation test diagram.

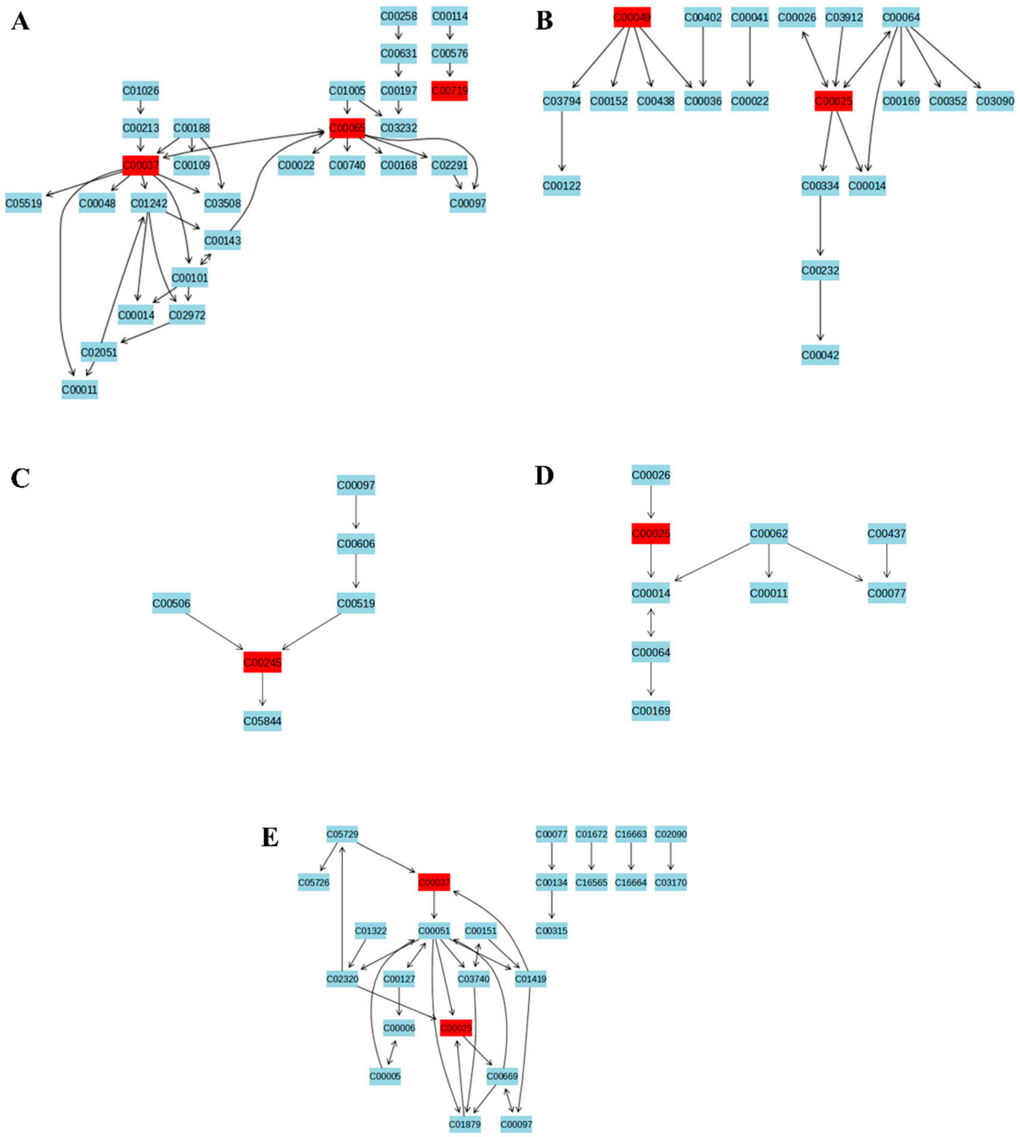

Fig. S6 The metabolic pathways: (A) Glycine, serine and threonine metabolism. (B) Alanine, aspartate and glutamate metabolism. (C) Taurine and hypotaurine metabolism. (D) Arginine biosynthesis. (E) Glutathione metabolism.

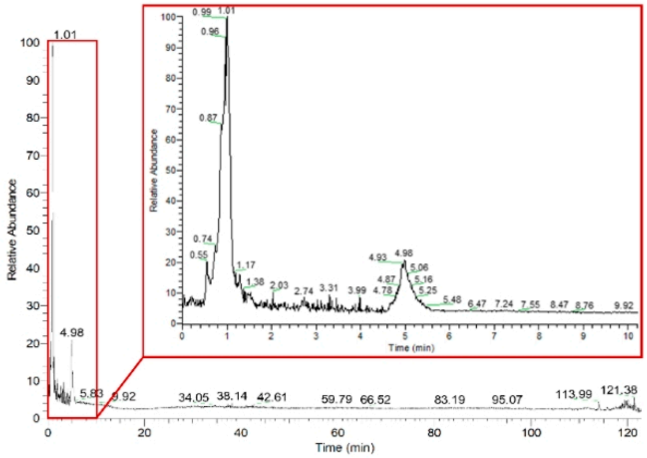

Fig. S7 Total ion current diagram of PMP. (Since the sample is a mixture of unclassified total bioactive peptides, the components are complex. Therefore, some components with similar polarity have co-elution on chromatography.)

Table S4 Peptide signal attribution.

NO.	Peptide	Tag length	ALC (%)	Length	RT	Mass
1	VLSAADKAVNK	11	99.8	11	0.718	1114.6346
2	HSCCCCH	7	98.4	7	5.3831	791.1971
3	VLSAADKGQVKA	12	89.2	12	0.7064	1185.6718
4	HDGEKGET	8	88.9	8	0.531	871.3671
5	GYCC	4	85.4	4	119.5879	444.1137
6	KKKL	4	85.1	4	119.4049	515.3795
7	HELNGGM	7	83.2	7	5.2937	756.3224
8	AKKK	4	82.9	4	118.732	473.3326
9	EHGDSFTLNHHDDH	14	75.9	14	0.5757	1659.6661
10	TQDDCC	6	74.5	6	93.7882	683.1891
11	KGVGANAPGCGHAALE	16	71.5	16	0.6943	1450.6986
12	AEEMCC	6	71.2	6	6.8943	684.1917
13	LPAALTK	7	69.9	7	0.954	712.4483
14	DDQTCC	6	69.8	6	43.5819	683.1891
15	HTAKKLLLALLLLH	14	68.6	14	5.5376	1583.0286
16	LVHK	4	68.5	4	97.3974	495.3169
17	PTLHL	5	68.4	5	0.8801	579.338
18	KGMVSATPPSL	11	67.8	11	0.7266	1086.5743
19	PPKGA	5	67.8	5	0.7521	468.2696
20	HDDGCMMYWCQQ	12	66.3	12	5.274	1515.504
21	TKLPVSL	7	65.8	7	0.9467	756.4745
22	KLLLLVTAVELHLH	14	65.8	14	111.1804	1597.9919
23	LDLP	4	65.7	4	110.5387	456.2584
24	VDDCTCC	7	65.3	7	20.5906	757.2081
25	LNVA	4	65.1	4	0.9504	415.2431
26	RVALPP	6	64.9	6	0.9221	651.4068

Table S4 Peptide signal attribution.

27	DVDCTCC	7	64.6	7	3.8332	757.2081
28	ASPCC	5	62.9	5	122.8494	479.1508
29	LVSAA	5	62.9	5	0.954	459.2693
30	ADACC	5	62.5	5	118.7197	481.1301
31	ASSSCC	6	62.3	6	117.293	556.1621
32	AEECTCC	7	62.1	7	2.1588	757.2081
33	TEDNCC	6	61.9	6	62.7408	683.1891
34	F	1	61.4	1	0.8906	165.079
35	W	1	61.3	1	0.9117	204.0899
36	KPPGP	5	61	5	0.7846	494.2852
37	PLAHKVPK	8	60.8	8	0.954	888.5545
38	CAANA	5	60.7	5	113.6604	448.174
39	KLRGLLLP	8	60.4	8	0.1726	908.6171
40	HDPKNVHVFPLYSP	14	59.9	14	5.04	1648.8362
41	VRPLPA	6	59.8	6	0.9221	651.4068
42	VKKKKKT	7	59.5	7	118.7898	858.6014
43	QVVPKGL	7	59.4	7	0.954	739.4592
44	KKPKRH	6	59.3	6	5.2826	792.5082
45	ATTSSTA	7	59.1	7	113.5836	637.2919
46	TMPCC	5	59.1	5	117.4083	553.1699
47	HDCCCCGCCCMCCHH	15	59.1	15	0.5085	1659.3589
48	R	1	58.4	1	117.3036	174.1117
49	CLDCC	5	58.3	5	121.3529	555.1491
50	QKLAPAL	7	58.1	7	0.954	739.4592
51	NESECC	6	57.9	6	97.1092	683.1891
52	CCVGPCC	7	57.6	7	3.0196	683.1899
53	AKATKPGA	8	57.5	8	0.9746	742.4337
54	KGPCAPG	7	57.4	7	0.8696	628.3002
55	KTAL	4	57.3	4	0.9435	431.2744
56	LDPTV	5	57.1	5	2.726	543.2904
57	VYPY	4	57	4	2.1177	540.2584
58	ETNDCC	6	57	6	58.7208	683.1891
59	AEDGSCC	7	56.8	7	44.1691	683.1891
60	VCCPGCC	7	56.8	7	51.9547	683.1899
61	GVCPCCC	7	56.7	7	23.5242	683.1899
62	QMCPCC	6	56.6	6	50.5266	683.1899
63	QNSCC	5	56.4	5	117.4083	553.1625
64	LCSDDCC	7	56.2	7	3.8332	757.2081
65	YFKK	4	56.2	4	1.3069	584.3322
66	ENESCC	6	56.1	6	3.0196	683.1891
67	AASDDCC	7	56	7	77.987	683.1891
68	HEGDHNFLGPDAATT	15	56	15	5.3737	1580.6855
69	CSDDLCC	7	55.9	7	17.4172	757.2081

Table S4 Peptide signal attribution.

70	TDNECC	6	55.9	6	93.5815	683.1891
71	NNTCTCC	7	55.8	7	39.2534	757.2194
72	NQSCTCC	7	55.6	7	13.55	757.2194
73	SSNGNCC	7	55.6	7	27.9484	683.2003
74	SDQECC	6	55.6	6	26.8473	683.1891
75	DETNCC	6	55.4	6	22.9739	683.1891
76	VVETP	5	55.4	5	4.9704	543.2904
77	LLAH	4	55.3	4	1.2292	452.2747
78	QCMPCC	6	55.3	6	20.2234	683.1899
79	SGWCTCC	7	55.1	7	6.65	758.2186
80	AMEECC	6	55	6	32.4679	684.1917
81	ATTCTE	6	55	6	116.8524	624.2425
82	NETDCC	6	54.9	6	0.3263	683.1891
83	LHVK	4	54.5	4	97.2178	495.3169
84	ENTDCC	6	54.5	6	16.5828	683.1891
85	PGDDGP	6	54.4	6	111.2862	556.2129
86	ACMGPCC	7	54.3	7	35.8578	683.1899
87	LQHVRLLL	8	54.2	8	97.2935	990.6338
88	GKLAATLK	8	54	8	0.933	800.512
89	ADDSACC	7	53.9	7	33.3984	683.1891
90	AHHKKVRL	8	53.7	8	42.1947	987.609
91	AYQMM	5	53.7	5	113.6206	642.2505
92	AHHALLKKA	9	53.7	9	87.1953	987.5978
93	VVCEEYFW	8	53.6	8	57.7125	1073.4528
94	DQDTCC	6	53.5	6	80.5581	683.1891
95	ARCCC	5	53.4	5	115.8936	554.1763
96	THYKPAVKN	9	53.4	9	0.7296	1056.5717
97	DNTECC	6	53.3	6	119.144	683.1891
98	ASDADCC	7	53.2	7	34.3399	683.1891
99	DQSECC	6	53.2	6	46.1106	683.1891
100	DENTCC	6	53.1	6	39.5216	683.1891
101	LPAAPLVRP	9	53	9	0.954	932.5807
102	AEFTMM	6	53	6	114.8164	728.2874
103	EENSCC	6	52.5	6	16.2153	683.1891
104	AWCC	4	52.5	4	122.8494	481.1454
105	QTDDCC	6	52.4	6	3.0196	683.1891
106	SENECC	6	52.3	6	59.0865	683.1891
107	RCCPCC	6	52.2	6	86.1288	683.2012
108	AHAKLRVPP	9	52.2	9	95.0777	987.5978
109	DTENCC	6	52.1	6	57.9211	683.1891
110	QPCMCC	6	52	6	90.6765	683.1899
111	NGNSSCC	7	51.9	7	51.1119	683.2003
112	TCGSWCC	7	51.8	7	5.9054	758.2186

Table S4 Peptide signal attribution.

113	DSEQCC	6	51.7	6	109.2159	683.1891
114	NSGATMM	7	51.7	7	118.0149	710.2728
115	GATDDCC	7	51.7	7	113.6195	683.1891
116	PMCQCC	6	51.5	6	100.5554	683.1899
117	NCCAAC	6	51.5	6	102.3606	583.1553
118	DDTQCC	6	51.3	6	75.7906	683.1891
119	PVCGCCC	7	51.3	7	27.2129	683.1899
120	GSDAECC	7	51.2	7	111.0182	683.1891
121	ACMPGCC	7	51.1	7	53.4589	683.1899
122	MPCQCC	6	50.9	6	46.6966	683.1899
123	PSVRT	5	50.9	5	14.6509	558.3126
124	KGFLLLLVTKGGPAAPW	17	50.7	17	0.6475	1767.0447
125	TCNSQCC	7	50.4	7	5.9533	757.2194
126	GKLLEVV	7	50.4	7	0.9467	756.4745
127	PHLAK	5	50.4	5	0.887	564.3384
128	ATDGDF	6	50.3	6	116.8524	624.2391
129	DNETCC	6	50.1	6	33.766	683.1891
130	TRHVT	5	50	5	0.8696	612.3344

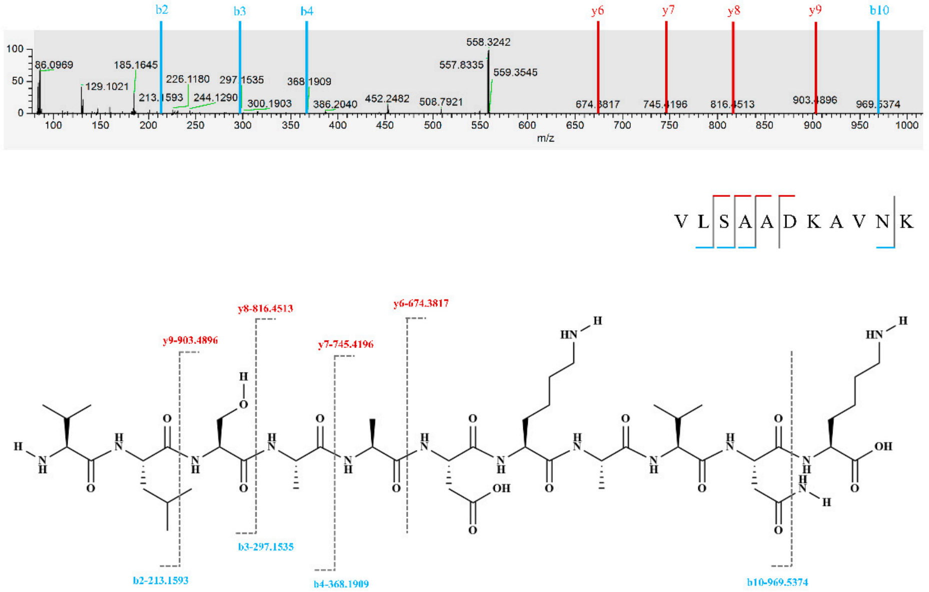

Fig. S8 Peptide sequence analysis based on MS/MS spectra: The MS/MS spectrum shows the b-ion ((N-terminal fragments: b2, b3-H_2O, b4-H_2O, b10) and y-ion fragments (C-terminal fragments: y6, y7, y8 y9) of VLSAADKAVNK. Immonium ions of Lys (m/z 86.0969) and Leu/Val (m/z 129.1021) further verified the amino acid composition.
